# Pan-Genome Portrait of Bacillus mycoides Provides Insights into the Species Ecology and Evolution

**DOI:** 10.1128/spectrum.00311-21

**Published:** 2021-07-21

**Authors:** Krzysztof Fiedoruk, Justyna M. Drewnowska, Jacques Mahillon, Monika Zambrzycka, Izabela Swiecicka

**Affiliations:** a Department of Microbiology, Medical University of Bialystok, Bialystok, Poland; b Department of Microbiology, Faculty of Biology, University of Bialystok, Bialystok, Poland; c Laboratory of Food and Environmental Microbiology, Earth and Life Institute, Université Catholique de Louvain, Louvain-la-Neuve, Belgium; d Laboratory of Applied Microbiology, Faculty of Biology, University of Bialystok, Bialystok, Poland; University of Minnesota

**Keywords:** *Bacillus mycoides*, pan-genome, plasmids, phages, insertion sequences, environment, adaptation, sigma factors

## Abstract

Bacillus mycoides is poorly known despite its frequent occurrence in a wide variety of environments. To provide direct insight into its ecology and evolutionary history, a comparative investigation of the species pan-genome and the functional gene categorization of 35 isolates obtained from soil samples from northeastern Poland was performed. The pan-genome of these isolates is composed of 20,175 genes and is characterized by a strong predominance of adaptive genes (∼83%), a significant amount of plasmid genes (∼37%), and a great contribution of prophages and insertion sequences. The pan-genome structure and phylodynamic studies had suggested a wide genomic diversity among the isolates, but no correlation between lineages and the bacillus origin was found. Nevertheless, the two B. mycoides populations, one from Białowieża National Park, the last European natural primeval forest with soil classified as organic, and the second from mineral soil samples taken in a farm in Jasienówka, a place with strong anthropogenic pressure, differ significantly in the frequency of genes encoding proteins enabling bacillus adaptation to specific stress conditions and production of a set of compounds, thus facilitating their colonization of various ecological niches. Furthermore, differences in the prevalence of essential stress sigma factors might be an important trail of this process. Due to these numerous adaptive genes, B. mycoides is able to quickly adapt to changing environmental conditions.

**IMPORTANCE** This research allows deeper understanding of the genetic organization of natural bacterial populations, specifically, Bacillus mycoides, a psychrotrophic member of the Bacillus cereus group that is widely distributed worldwide, especially in areas with continental cold climates. These thorough analyses made it possible to describe, for the first time, the B. mycoides pan-genome, phylogenetic relationship within this species, and the mechanisms behind the species ecology and evolutionary history. Our study indicates a set of functional properties and adaptive genes, in particular, those encoding sigma factors, associated with B. mycoides acclimatization to specific ecological niches and changing environmental conditions.

## INTRODUCTION

The Bacillus cereus sensu lato encompasses endospore-forming aerobic rods present in a wide variety of habitats, including soil, water, and plant and animal tissues as well as food matrices (1–3). These Gram-positive bacilli display various forms of lifestyles, from saprophytic soil bacteria to intestinal symbionts of insects and mammals or to human pathogens (2, 4). Due to specific features such as endospore and/or toxin production, members of the B. cereus group have significant impacts on human health, agriculture, and food qualities. For instance, infection with Bacillus anthracis, the etiological agent of anthrax, can result in high mortality of humans and herbivores (5). Also, some strains of B. cereus sensu stricto and Bacillus cytotoxicus, recognized as saprophytes or opportunistic foodborne pathogens, are occasionally involved in food poisoning and intestinal infections associated with a variety of toxins (6, 7). However, Bacillus thuringiensis, an insect pathogen, has remarkable biotechnological potential, as it is used as a biopesticide against numerous crop pests or to control vectors of animal diseases (8). Similarly, Bacillus toyonensis is exploited as a probiotic in animal feed (9). These bacteria still generate growing interest, for both scientific and economic reasons. This is in contrast with Bacillus mycoides, another member of the B. cereus group, which has so far remained much less studied, although it displays several interesting features, including adaptation to low temperature.

Due to recent reclassification within B. cereus sensu lato (10), B. mycoides encompasses psychrotrophic bacilli forming unusual rhizoidal colonies on agar plates and Bacillus weihenstephanensis forming regular colonies on solid media. The latter was distinguished from B. cereus based on its psychrotrophic character and the presence of specific signatures in the *cspA* and 16S rRNA genes (11). Yet, several studies have since suggested to merge these two species. For instance, Liu and coworkers (12) gathered B. weihenstephanensis and B. mycoides strains in one group with similarity of approximately 80% in the digital DNA-DNA hybridization (dDDH), while Drewnowska and Swiecicka (13) noted that isolates classified as these taxa fell into the same phylogenetic clade. Based on multifaceted analyses (e.g., dDDH, metabolic activities, physiological features, and chemotaxonomic traits), it was recommended to consider B. weihenstephanensis as a heterotypic synonym of B. mycoides (10). In fact, psychrotolerance and specific sequences in the 16S rRNA genes and *cspA* genes did not properly distinguish B. weihenstephanensis from some other B. cereus sensu lato (14). Based on this reclassification, in this work, we consider as B. mycoides both the isolates of rhizoidal colonies and those forming regular colonies, which have the ability to grow at temperatures ranging from 5 to 40°C. Adaptation to temperatures is one of the most important factors of bacterial diversification (15). B. cereus sensu lato comprises bacilli growing at temperatures ranging from psychrotrophic (B. mycoides and B. wiedmannii) through mesophilic (B. anthracis, B. cereus sensu stricto, and B. thuringiensis) to thermophilic (*B. cytotoxicus*) (4, 16). Moreover, thermotypes within the group adapted to different thermal niches are congruent with their ribosomal protein profiles (17) and the phylogenetic groups I to VII (7, 15). With regard to psychrotrophic species, while *B. wiedmannii* belongs to phylogroup II, B. mycoides (together with former B. weihenstephanensis) constitutes phylogroup VI in the scheme proposed by Drewnowska et al. (7) and Guinebretière et al. (15).

Strains of our B. mycoides collection have been isolated from soil samples of continental cold climate areas according to the Köppen-Geiger climate classification (7). Interestingly, it was noted that the B. mycoides isolates differed in their capacity to utilize chitin (18) and to synthesize water-soluble melanin (19). Nevertheless, little is known about the ecology and adaptation of B. mycoides to different habitats or the extent of species diversity, especially from a genomic standpoint. It seems that B. mycoides plays important roles in the functioning of natural environments, and some genes present in its genome can be transferred between members of the group via, *inter alia*, the conjugation processes, as observed between other members of B. cereus sensu lato (20, 21). Factually, B. cereus sensu lato is characterized by intensive genome plasticity. Plasmids, phages, and other genetic elements including insertion sequences (ISs) may constitute a significant part of their genomes (22–24). These genetic elements are, at the same time, involved in B. cereus sensu lato adaptation to diverse environments and species evolution mostly through mutation, recombination, or horizontal gene transfer (25, 26).

The development of next-generation sequencing (NGS) technologies has enriched public databases with numerous sequenced genomes, making it possible to expand the understanding of intra- and interspecies genome diversity and bacterial evolution. Especially, comparative genomic studies with the use the pan-genome concept allow us to assess the genome modification and provide insights into bacterial evolution and adaptation to specific environmental niches (27–31). To obtain insight into B. mycoides biology and evolutionary history, we performed a deep comparative investigation of the B. mycoides pan-genome and the functional gene categorization of isolates originating from environments differing in nutrient status and anthropogenic pressure.

## RESULTS

### Genome features of B. mycoides from northeastern Poland.

The B. mycoides isolates under study were isolated from soil samples taken in northeastern Poland. Twenty-one originated from Białowieża National Park (BPN), the last European natural primeval forest under protection of World Heritage as a biosphere reserve and with soil classified as organic, while 14 strains were from mineral soil samples taken from a farm in Jasienówka (JAS), a place with strong anthropogenic pressure. Their genomes were sequenced using next-generation sequencing (NGS). The main features of the genomes and the National Center for Biotechnology Information (NCBI) database accession numbers are shown in Table S1 in the supplemental material, whereas detailed characteristics of individual isolates, such as (i) chromosomes, plasmids, and genome length, (ii) the number of coding DNA sequences (CDSs), (iii) the number of various types of RNAs, and (iv) GC content, are summarized in Table S2.

**FIG 1:**
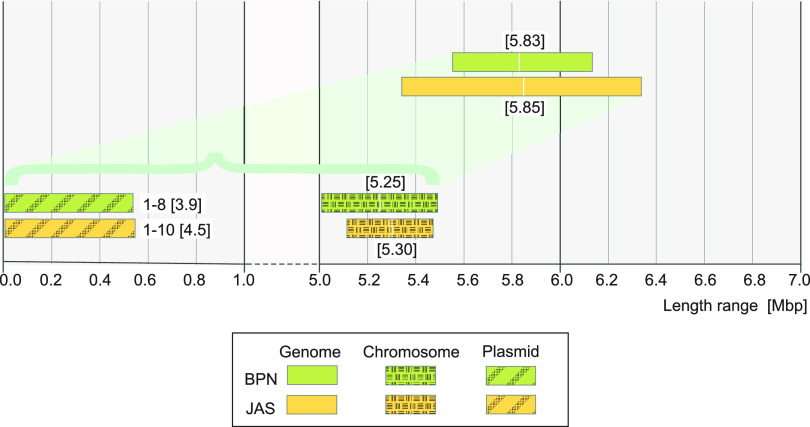
Characteristics of genomes of B. mycoides isolated from soil samples collected in Białowieża National Park (BPN; green plots) and in a farm in Jasienówka (JAS; yellow plots), northeastern Poland. Average length of genomes and chromosomes are given in brackets. The range of the plasmid number and average number of plasmids per strain are shown next to the plasmid range size plots.

The average lengths of B. mycoides genomes were similar in both populations, but there was a slightly narrower range among the BPN isolates (from 5.55 to 6.13 Mb) than among the farm ones (from 5.34 to 6.34 Mb) (Fig. 1; Table S3). Both fit into the genome size range of the species reference strains, which varies from 4.88 to 6.50 Mb (NCBI). Plasmid lengths ranged from 3.78 to 538.90 kb and from 3.95 to 545.96 kb within the BPN and JAS populations, respectively (Fig. 1; Tables S2 and S3). The mean GC contents of the genomes were 33.8% (BPN isolates) and 33.6% (JAS isolates), while the number of CDSs varied from 5,463 to 6,466 among the BPN isolates and from 5,296 to 6,368 among the JAS isolates (Table S2).

### B. mycoides pan-genome.

**FIG 2:**
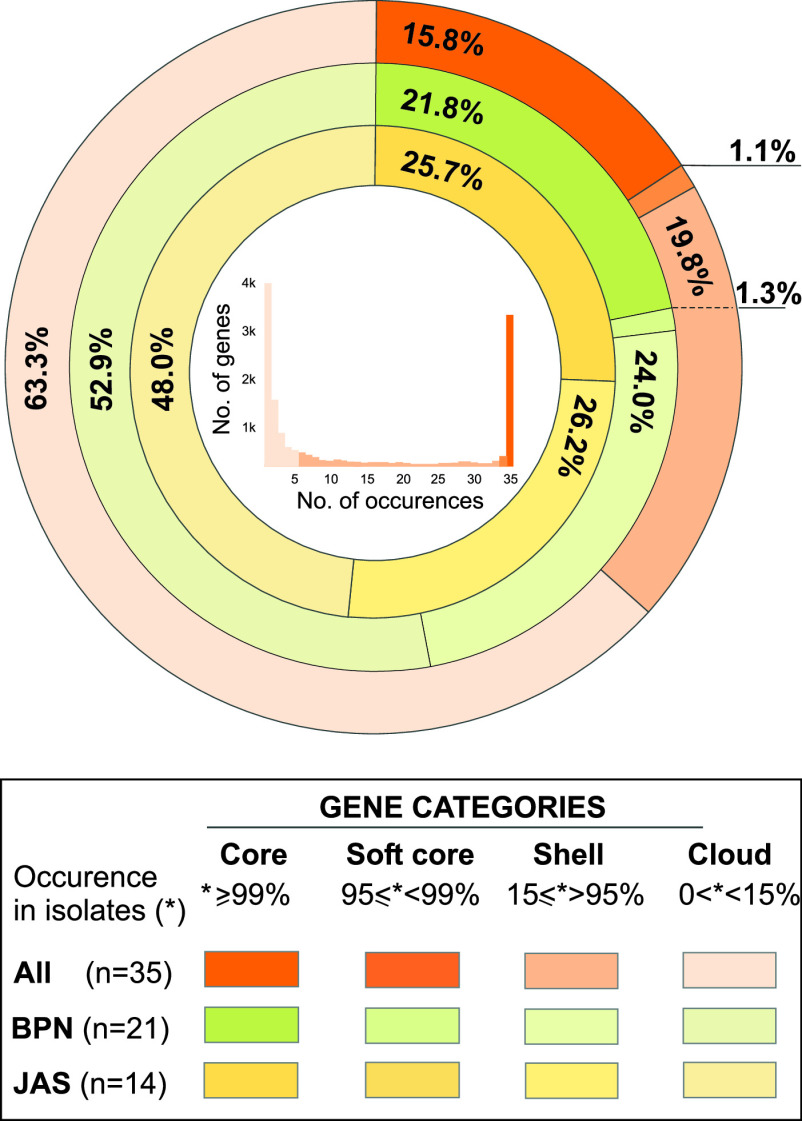
Percentages of genome genes in each pan-genome category for all B. mycoides under study (orange ring), isolates from Białowieża National Park (BPN; green ring), and isolates from a farm in Jasienówka (yellow ring). The distribution of gene occurrences in B. mycoides genomes is shown in the graph in the center of the figure, where genes present in one genome only are indicated by the left peak, and those present in all genomes are indicated by the right peak.

A pan-genome, considered all genes occurring in the analyzed data set, is generally divided into four categories of genes (core, soft core, shell, and cloud) based on their presence in the analyzed set of strains. The last two gene categories are considered the adaptive genes. As shown in Table 1 and Fig. 2, the B. mycoides pan-genome is complex, with a predominance of adaptive genes that account for up to 83.1%.

**TABLE 1:** Pan-genome and pan-plasmidome of B. mycoides isolates from northeastern Poland

Category	No. of genes					
	Pan-genome^*a*^			Pan-plasmidome^*b*^		
	BPN (*n* = 21)	JAS (*n* = 14)	All isolates (*n* = 35)	BPN (*n* = 21)	JAS (*n* = 14)	All isolates (*n* = 35)
Core genes	3,409	3,526	3,185	4	0	0
Soft-core genes	201	0	201	0	0	0
Shell genes	3,738	3,597	4,008	859	877	827
Cloud genes	8,254	6,608	12,781	4,070	3,214	6,564
Total genes	15,602	13,731	20,175	4,933	4,091	7,391

a Core genes are common to at least 99% of strains; soft-core genes are present in between 95% and 99% of strains; shell genes are present in between 15% and 95% of strains; cloud genes are present in less than 15% of strains; total genes, a collection of all genes present in the whole set of strains under study.

b Core genes, genes present on plasmids harbored by at least 99% of strains; soft-core genes, genes present on plasmids harbored by between 95% and 99% of strains; shell genes, genes present on plasmids harbored by between 15% and 95% of strains; cloud genes, genes present on plasmids harbored by less than 15% of strains; total genes, a collection of all genes present on plasmids harbored by the whole set of strains under study.

**FIG 3:**
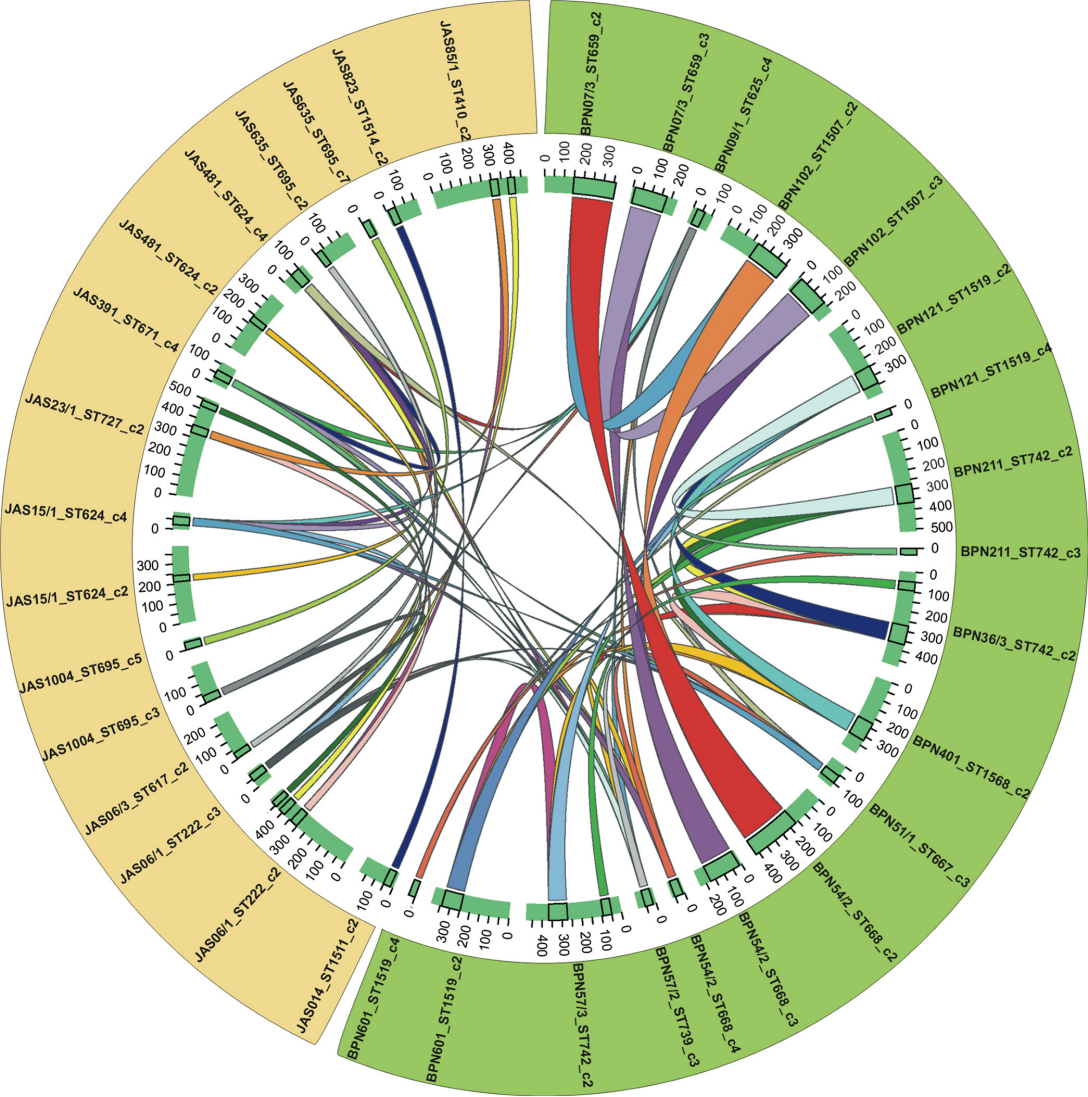
Nucleotide sequence-based comparison of 30-kb synteny DNA fragments of plasmids harbored by B. mycoides under study. Plasmids present in the isolates from Białowieża National Park (BPN) are marked in green, while those from the Jasienówka farm (JAS) are marked in yellow. ST, sequence type; c2, c3, c4, and c5, contigs indicating plasmids in particular strains (for details, see Table S2 in the supplemental material).

The *in silico* analysis detected 146 plasmids among the 35 B. mycoides isolates, contributing from 1.50% to 14.68% of their genomes (Fig. 1; Table S2). The presence of plasmids smaller than 100 kb was confirmed by their separation through agarose gel (Fig. S1). In the B. mycoides under study, 7,391 genes were found to be associated with plasmids (*n* = 146). These genes were divided into the pan-plasmidome categories by extending the concept of the pan-genome (Table 1). Genes meeting the conditions of the core and soft-core pan-plasmidome were not found, yet 827 genes were classified into the shell category and 6,564 were classified as cloud genes. Interestingly, in the BPN plasmids, but not in the JAS ones, four genes were found to be common to all plasmids (Table 1). Nevertheless, a significant majority of the plasmid genes, 4,070 (82.5%) and 3,214 (78.6%) within BPN and JAS B. mycoides, respectively, occurred on plasmids harbored by less than 15% of the isolates under study. Plasmid comparison revealed the presence of common DNA fragments much more often between plasmids found among isolates of the same population, especially in those originating from Białowieża National Park (Fig. 3).

**FIG 4:**
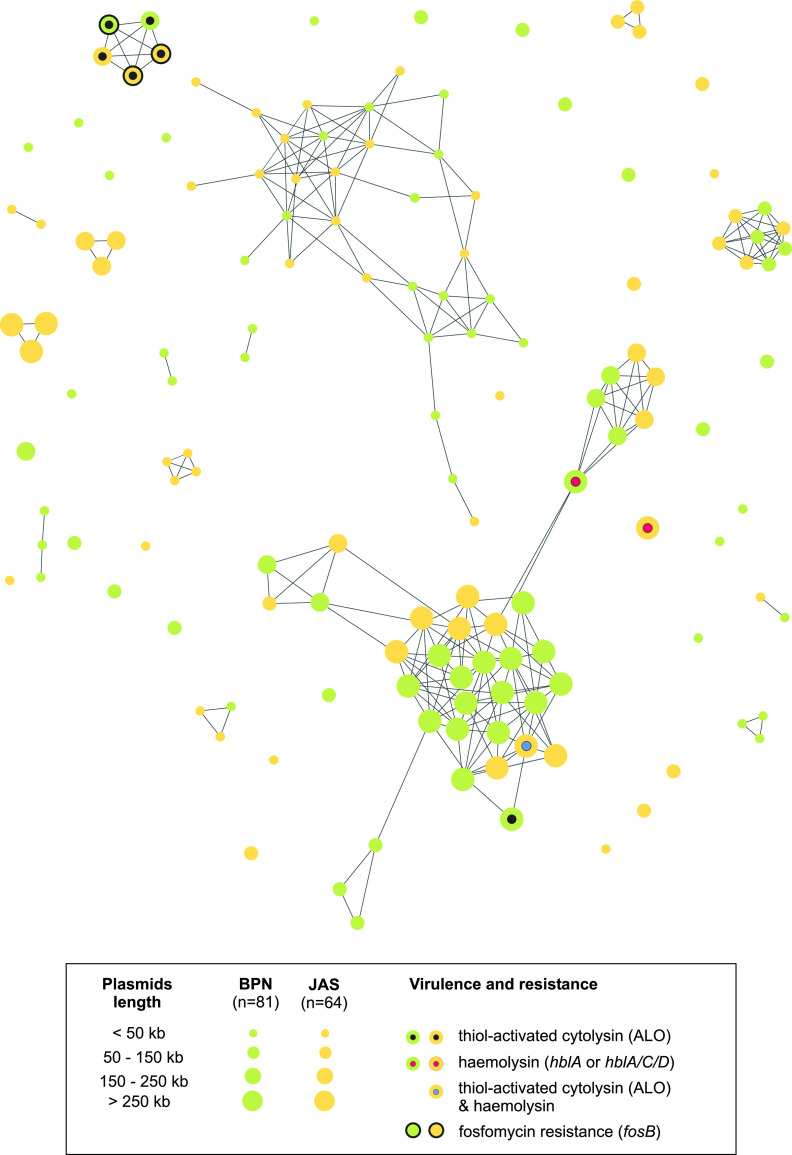
Nucleotide sequence-based similarity network of B. mycoides plasmids from soil samples from Białowieża National Park (green circles) and a farm in Jasienówka (yellow circles) achieved with the plasmid ATLAS (pATLAS) program.

In the similarity network achieved with the plasmid ATLAS (pATLAS) platform, two large clusters comprising plasmids harbored by isolates from both locations were observed (Fig. 4). One cluster was composed of 31 small plasmids (<50 kb), and the second one was composed of 38 plasmids mostly of a size >250 kb, which formed four subclusters. The other plasmids (*n* = 76) were singletons or clustered in small assemblages, localized separately from the above-mentioned large clusters. Note that nine B. mycoides plasmids were found to be potentially involved in toxinogenesis (encoding thiol-activated cytolysin and/or hemolysin BL) and fosfomycin resistance. Five of these plasmids, all encoding thiol-activated cytolysin, formed a single cluster (Fig. 4).

**FIG 5:**
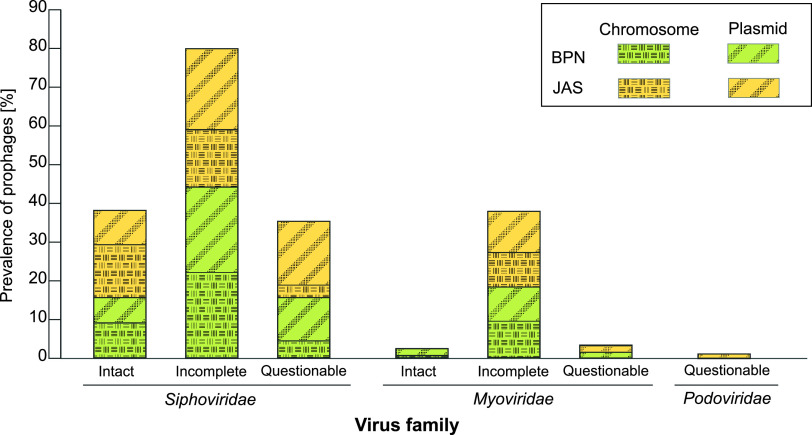
Prevalences of prophages among B. mycoides populations from northeastern Poland. The prediction of whether the region contains a prophage was achieved with the PHASTER web server (accessed June 2020) based on a completeness score as follows: an intact phage, score >90%; an incomplete phage, score 70% to 90%; a questionable phage, score <70%.

Besides plasmids, prophages are of the highest significance in lateral gene exchange in bacteria. Altogether in the genomes of 35 B. mycoides isolates under study, we found 37 putative, 35 questionable, and 108 incomplete prophages (Table S4). An important proportion of these elements (59%) were located on plasmids. The prophages were classified to *Siphoviridae*, *Myoviridae*, and *Podoviridae*, with the predominance of the first family in both B. mycoides populations (Fig. 5). Detailed characteristics of prophages present in the B. mycoides isolates, e.g., prophage location, region length, total number of proteins encoded, GC content, and the most common phage corresponding to the sequence, are given in Table S4.

**FIG 6:**
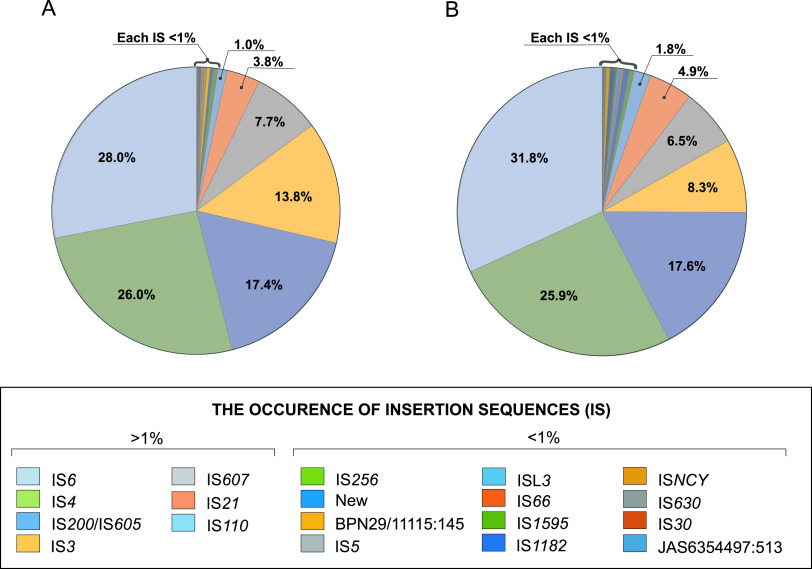
Occurrences of insertion sequences (ISs) in genomes of B. mycoides originating from Białowieża National Park (A) and a farm in Jasienówka (B).

To improve on the understanding of the B. mycoides genome organization, we performed a thorough *in silico* analysis of the IS occurrence in Polish isolates of the species. Altogether, 1,381 and 921 potential ISs were found within the BPN and JAS populations, respectively, with the number in individual isolates ranging from 44 (BPN29/1) to 119 (BPN03/1) among BPN isolates and from 37 (JAS94/5) to 119 (JAS83/3) among JAS isolates (Table S5). ISs present in B. mycoides isolated from soil samples taken in Białowieża National Park were classified into 16 families (Fig. 6). With the exception of IS*30*, all of these families were also noted in the genomes of JAS isolates. In B. mycoides from both locations, members of the IS*6* (∼30%), IS*4* (∼26%), and IS*200*_IS*605* (∼17%) predominated (Fig. 6).

### Phylodynamic studies of B. mycoides.

In a phylogenetic tree constructed using a maximum likelihood method from the aligned single nucleotide polymorphism (SNP) positions in chromosomes, isolates under study grouped together with three B. mycoides reference strains, whereas the other nine references (eight representing B. cereus sensu lato and Bacillus subtilis 168) clustered outside B. mycoides (Fig. S2). These results strongly indicate the monophyletic nature of the *Bacilli* examined. Interestingly, subclusters within B. mycoides included isolates from both populations. In the average nucleotide identity (ANI) analysis, performed in order to clarify the genetic relatedness between B. mycoides, the monophyletic nature of the isolates and the B. mycoides references was confirmed (Fig. S3). The ANI values ranged from 94.89% (BPN601 versus JAS014) to 99.98% (BPN121 versus BPN601) among isolates and from 94.90% (DSM11821 versus JAS014) to 99.51% (ATCC 6462 versus JAS48/1) when the B. mycoides references strains were considered. As expected, the topologies of both the phylogenetic tree (Fig. S2) and the ANI tree (Fig. S3) were similar, i.e., the isolates closely related in the phylogenetic tree fell into the same ANI assemblages, such as in the case of BPN121, BPN211, BPN36/3, BPN573, BPN601, and BPN37/2.

**FIG 7:**
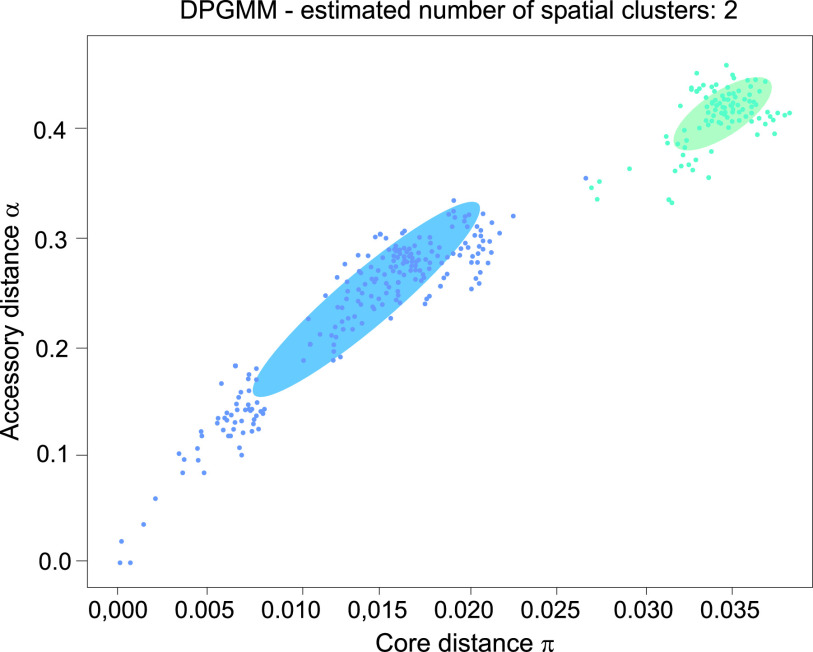
The distribution of core (π, *x* axis) and accessory (*a*, *y* axis) pairwise genome distances of B. mycoides under study. The distances were calculated using Dirichlet process Gaussian mixture Model (DPGMM) in the PopPUNK software.

The distribution of core and accessory pairwise chromosome distances with PopPUNK showed the presence of two clusters within B. mycoides, each containing both BPN and JAS isolates (Fig. 7; Table S2). The 28 bacilli forming the first cluster (a blue ellipse in Fig. 7) are more similar in both their core (*a*) and accessory (π) genomes (*a* ranged from 0.15 to 0.32; π ranged from 0.006 to 0.020), compared to the second one (a turquoise ellipse) comprising seven isolates (*a* ranged from 0.38 to 0.48; π ranged from 0.032 to 0.039).

### Functional characterization of B. mycoides genes.

The classification against Kyoto Encyclopedia of Genes and Genomes (KEGG) with the use of the BlastKOALA algorithm (32), summarized in Table S7, was achieved for 106,028 (61.8%) of 171,469 and 6,445 (45.5%) of 14,196 chromosomal and plasmid annotated proteins, respectively. In B. mycoides from both locations, the major portions of genes were associated with metabolism, with a predominance of carbohydrate metabolism, amino acid metabolism, and metabolism of cofactors and vitamins among both the chromosomal and plasmid genes. The second largest number of orthologs pertained to the environmental information processing category. In both populations, this group encompassed around one-fourth of the chromosomal genes and even higher proportions of the plasmid genes (specifically, 27% and 32% of plasmid genes in the BPN and JAS bacilli, respectively). The genes grouped in the genetic information processing made the third largest group, with a predominance of the translation category among the chromosomal genes and replication/repair among the plasmid genes.

**FIG 8:**
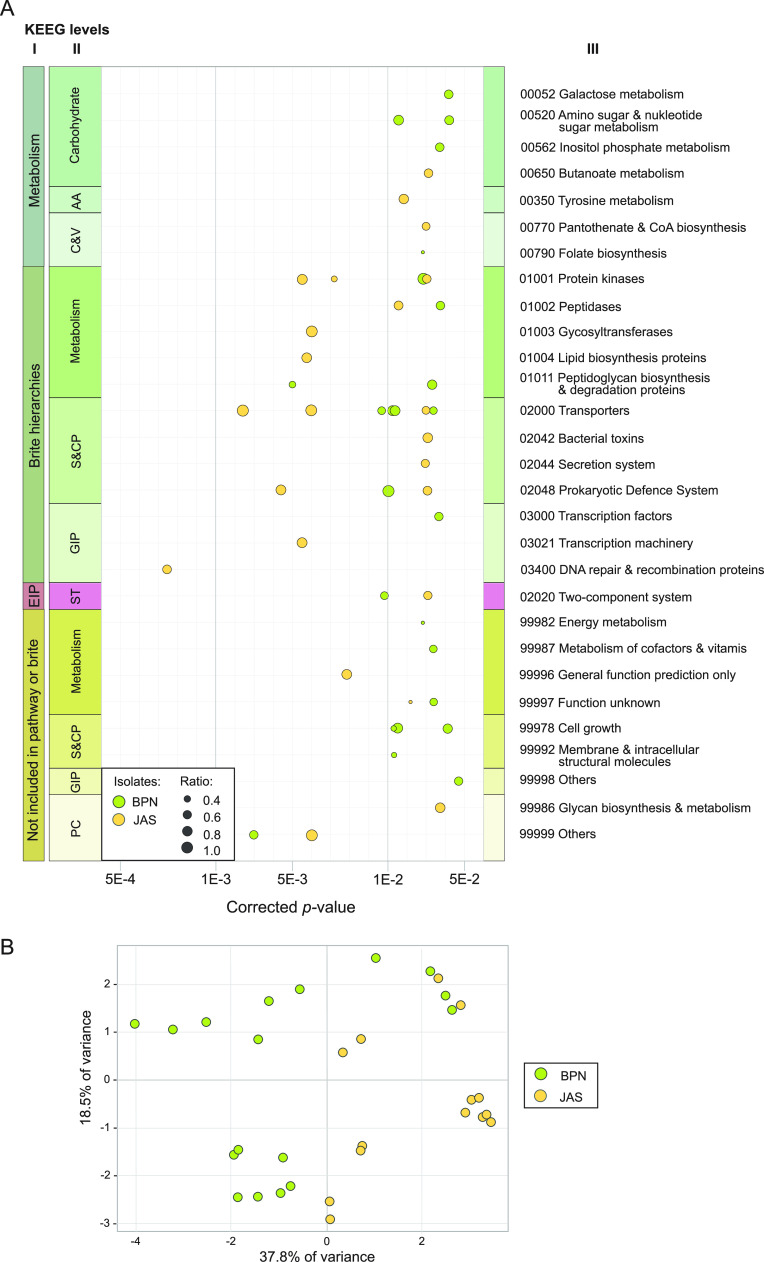
Significant differences in the presence of the KEGG level 3 categories in B. mycoides from Białowieża National Park (BPN) and the Jasienówka farm (JAS). (A) Differences assessed based on Fisher exact test (*P* < 0.05). (B) Principal-component analysis (PCA) of the statistically significant differences in the presence of the level 3 KEGG categories in the two populations. Ratio indicates the quotient of the number of strains carrying a gene to the number of strains in the population. Isolates belonging to each population are identified in green (BPN) and yellow (JAS).

Although the proportion of the KEGG functional level 2 categories among B. mycoides populations originating from Białowieża National Park and Jasienówka farm were similar (Table S7), significant differences between these two populations were found within the KEGG level 3 categories (Fig. 8). For instance, 52.4% of BPN isolates (versus 14.3% of JAS isolates) carried a gene coding the mycobilin-producing heme oxygenase (EC 1.14.99.57), and 61.2% of them (versus 21.4% of JAS isolates) possessed an operon responsible for *myo*-inositol utilization. Meanwhile, most JAS isolates (85.7%) and only 33.3% of BPN isolates possessed the *sigB* operon (Fig. S4), and the majority of them (71.4%) possessed loci carrying genes associated with lantibiotic synthesis; this feature seems to be less common within the BPN population (19.0%). On the other hand, an operon encoding alternative σ^70^ family RNA polymerase sigma factor (denoted *sigM-3*) seems to be unique for BPN isolates (71.4%) (Fig. S5). In addition, 64.3% of the JAS isolates (versus 4.8% of BPN isolates) carried the DNase V gene (EC 3.1.21.7).

## DISCUSSION

Prokaryotes are characterized by immense changeability, mostly associated with (i) episodic mutations, (ii) transposition, (iii) horizontal gene transfer (HGT), especially through transformation, transduction, and conjugation, and (iv) gene loss events, which are not as noticeable as gene acquisition via HGT. The balance between these processes reveals a definite shape of gene occurrence in prokaryote genomes. As a result, huge variations in gene content can be observed within species or even within clones or lineages (33). While the core and the soft-core genes encode proteins associated with fundamental biological processes and phenotypic features, adaptive genes significantly contribute to the species diversity by encoding supplementary biochemical pathways and additional functions, mostly essential for selective advantages to different niches (27, 34). The predominance of adaptive genes (83.1%) in the B. mycoides pan-genome allows these bacilli to inhabit various ecological niches and to function in a variety of environments.

The differentiation of bacterial pan-genomes is closely related to the species lifestyle (29) as well as to genome features, such as the ability to integrate exogenous DNA (34, 35). For instance, in obligate intracellular pathogens, such as Piscirickettsia salmonis causing salmonid rickettsial septicemia (36) and Chlamydia spp. involved in pneumonia infections (37), the core genes constitute approximately 50% of their pan-genomes. In contrast, members of the B. cereus group display smaller core genomes, e.g., 31% for B. cereus (38), 39% for B. thuringiensis (38), and 16% for B. mycoides (this study), indicating a much higher potential for adaptation. Interestingly though, B. anthracis, the most pathogenic member of the B. cereus group, also recognized as a monophyletic species (5), has a core genome that reaches 64% of its pan-genome (38).

B. cereus sensu lato plasmids can make up a large part of genomes and play crucial roles in many processes linked to secondary metabolism, virulence, and environmental adaptation (22, 25, 39, 40). However, whereas plasmids in B. cereus sensu lato species known for pathogenicity, such as B. anthracis or B. thuringiensis, have been extensively studied (8, 24, 26, 40), knowledge about these genetic elements in the other members of the group, such as those harbored by B. mycoides, remains limited. Our study showed a richness of genes associated with B. mycoides plasmids, as all of them represented the adaptive pan-plasmidome category. Interestingly, the presence of common DNA fragments among plasmids found from isolates of the same population suggests that horizontal gene transfer (HGT) may occur frequently among *Bacilli* classified to the species. Recently, Hu et al. (21) evidenced that in soil, B. thuringiensis plasmids with conjugal and mobilizing capability provoke gene transfer within the B. cereus group populations and, in consequence, potentially modify the population structure of these bacilli. Thus, a similar process cannot be excluded with regard to B. mycoides, especially in soil rich in nutrients, as it was noted among the Białowieża National Park samples. Similarly, the B. mycoides plasmids were found to be potentially involved in intestinal infection and antibiotic resistance. Moreover, as for other B. cereus sensu lato members, B. mycoides harbors prophages on both chromosomes and plasmids. It is worth noting that these prophages contained a significant number of CDSs (3,685 and 2,360 within BPN and JAS isolates, respectively), which may be of great importance for the functioning of these bacilli in diverse ecological niches and their evolution.

Differentiation among species of the B. cereus group mostly results from lateral gene transfer, intragenomic rearrangement within genomes, and the ability to integrate in genomes as exogenous DNA. These processes are mostly linked to insertion sequences (ISs), which are common in prokaryotic genomes and have the capacity to import many genes into bacteria over short times (25, 26, 41). ISs are considered the main actors in evolutionary dynamics within bacteria due to their involvement in various DNA rearrangements, including insertions and deletions (so-called indel events), duplications, and inversions (32). For instance, indel events were responsible for the disruption of the *sigM-3* operon in JAS isolates (Fig. S5). As with other bacterial species, ISs have likely affected genome plasticity displayed by B. cereus sensu lato (25). In the B. mycoides genomes under study, IS*6*, IS*4*, and IS*200*_IS*605* predominated, which were also indicated by Fayad et al. (25) in genomes of four B. mycoides strains and two B. weihenstephanensis strains. These authors observed variability of the IS distribution among B. cereus sensu lato species and concluded that some families such IS*3*, IS*4*, and IS*200*/IS*605* are ubiquitous among members of the group, whereas others such as IS*1*, IS*30*, and IS*982* were seldom, which is in line with our observations of the 35 B. mycoides isolates. It is noteworthy that IS*3*, IS*4*, and IS*6* are also dominant in genomes of B. thuringiensis serovar israelensis (26). Such a numerous representation of ISs in B. cereus sensu lato gives these bacilli additional possibilities of dynamic changes of their genomes, successfully adapting these bacteria to the occupied environments. Indeed, colocalization of disrupted IS*Bth5* element (IS*4* family) possibly explains the uneven distribution of the *sigB* operon between BPN and JAS populations (see Fig. S5 in the supplemental material).

Sigma factors, in particular, the extracytoplasmic function (ECF) family, owing to their fine-tune regulation of stress response genes, are considered key elements of adaptation to specific environmental changes and factors shaping bacterial evolution (42). For instance, duplication (or loss) of sigma factor genes followed by their further divergence and specialization in controlling of a smaller number of genes, e.g., those critical for the adaptation and survival in new ecological niches, was proposed by Schmidt et al. (43) as an important driving force behind the evolution of B. cereus sensu lato members. More importantly, such subfunctionalization of sigma factors may subsequently lead to divergence/specialization of the regulated genes. Therefore, it will be interesting to investigate whether the differences in SigB and SigM-3 prevalence between BPN and JAS isolates is correlated with differential regulation. Although B. cereus SigB was recognized as an important heat shock protein (HSP) from group II (i.e., with maximum induction after 15 min, which also involves factors activated by other stresses, such as salt or ethanol [44]), the function of SigM-3 remains to be elucidated. Nevertheless, from the evolutionary perspective, SigM-3 may represent a primal adaptation to the unique environment of the natural primeval forest, since it represents a different type of sigma factor (Fig. S6) and seems to be less redundant in JAS isolates.

In the PopPUNK analysis, the B. mycoides strains under study were classified into two clusters. Interestingly, in the “blue” cluster (with lower values of the *a* and π parameters) the average IS count was smaller than for the bacteria of the “turquoise” group (with higher values of the *a* and π parameters). Although the low number of bacilli studied does not allow any final conclusions to be drawn, these results suggest that among mobile genetic elements (MGEs), ISs play an important role in genetic variation of B. mycoides, as was also earlier observed for other members of the group, especially B. thuringiensis and B. cereus sensu stricto (25, 45).

The ANI analysis brought strong evidence that the isolates under study belong to B. mycoides according to a scheme proposed by Carroll et al. (46). Yet, in both the B. mycoides phylogenetic tree and the ANI heat map, the isolates displaying rhizoidal growth and those forming regular colonies on solid media occurred in the same clades within the phylogenetic tree, proving additional legitimacy of the B. mycoides and B. weihenstephanensis reclassification into one species within the B. cereus group (10). Moreover, although some BPN and JAS B. mycoides isolates showed the tendency to group together, there was no a clear distinction between bacilli originating from the two locations, i.e., Białowieża National Park and the Jasienówka farm.

Next-generation sequencing of bacterial isolates and the search for functional categories of B. mycoides genes allowed us, on one hand, to portray the ortholog content of these bacilli and, on the other hand, to report functional differences among the pan-genomes of isolates originating from diverse environments (33). In the KEGG classification accomplished in this study, the major portions of genes were associated with metabolism, environmental information processing, and genetic information processing. Interestingly, a large proportion of plasmid genes in B. mycoides was sorted into the replication/repair category. Indeed, a substantial portion of genes from this category was earlier observed in the accessory genomes of other B. cereus sensu lato isolates and in the B. subtilis group by Kim et al. (38). The authors suggested that these genes are often engaged in mobilome-related processes (38), which has a significant role in the evolution of this species (25).

B. mycoides from Białowieża National Park and Jasienówka farm differed significantly in the presence of genes encoding specific proteins enhancing these bacilli in their natural environments. For instance, BPN isolates can use inositol as a carbon source due to the presence of the operon responsible for stepwise reactions of *myo*-inositol. Indeed, this sugar cyclic polyalcohol is common in soil and plants (47, 48), which may suggest that the *myo-*inositol degradation operon gives environmental advantages to occupy niches where this compound is present as a source of carbon (47). Along the same lines, most JAS isolates (i) can synthetize lantibiotic, as it was also observed among other members of the B. cereus group (49), and (ii) possess DNase V (EC 3.1.21.7), providing an alternative repair mechanism for DNA damage caused by, *inter alia*, environmental factors such as nitric oxide free radicals (50). Moreover, similarly to other representatives of the *Bacillus* genus, such as the B. subtilis group (51), B. mycoides and other members of B. cereus sensu lato produce a set of compounds (52, 53) facilitating the colonization of various ecological niches.

In conclusion, in this study, we mainly focused on the identification of the B. mycoides pan-genome and its functional properties associated with adaptation of these bacilli to specific ecological niches. Thanks to this study, the number of genomes available for this species increased from 74 to 109, constituting a valuable data set for comparative analyses undertaken for both the B. mycoides species and the whole B. cereus group. Soil, the primary niche for B. mycoides and its relatives in the B. cereus group (2, 13), is a highly heterogenous environment, in which the content of nutrients, particle size, humidity, and chemical properties may change drastically in short space and time frames (21, 54). The results of the present study illustrate how B. mycoides and kin have and are well adapted to these ever-changing environments.

## MATERIALS AND METHODS

### B. mycoides isolates.

Altogether, we studied 35 B. mycoidesisolatesfrom soilsamples takenin highly diverse environments, e.g., in the natural reserve of Białowieża National Park (*n* = 21; BPN isolates) and a farm in Jasienówka (*n* = 14; JAS isolates), both located in northeastern Poland (see Table S1 in the supplemental material). Bacterial isolation, multilocus sequence typing (MLST) (https://pubmlst.org/organisms/bacillus-cereus), and soil sample examination were performed during a previous project (13). For this study, isolates were taken randomly from a set of B. mycoides preliminary characterized by MLST as mentioned above (13). With a few exceptions, the isolates represented different sequence types (STs): among the 21 BPN isolates, 17 had different STs, and the 14 JAS isolates belonged to 12 STs (for details see Table S1).

### Next-generation sequencing.

Genomic DNA of the B. mycoides isolates was extracted from an overnight culture of an isolate in Luria-Bertani (LB) broth using a DNeasy blood and tissue kit (Qiagen GmbH, Hilden, Germany) and the manufacturer’s protocol for Gram-positive bacteria. The DNA quality was assessed using a NanoDrop 2000 spectrophotometer (Thermo Fisher Scientific, Wilmington, DE, USA) and gel electrophoresis, while the DNA quantity was measured using both a Qubit 2.0 fluorometer with a Qubit double-stranded DNA (dsDNA) HS assay kit (Thermo Fisher Scientific) and a 2200 TapeStation instrument with a genomic DNA ScreenTape assay (Agilent Technologies Inc., Santa Clara, CA, USA).

Each isolate was sequenced using both Illumina (Illumina Inc., San Diego, CA, USA) and Oxford Nanopore Technologies (ONT; Oxford, United Kingdom) sequencing platforms. For Illumina, genomic libraries were prepared using a Nextera XT Index kit (Illumina Inc.) according to the manufacturer’s protocol (document no. 15031942 v02) and quantified by capillary electrophoresis, applying the Agilent high-sensitivity D5000 ScreenTape system (Agilent Technologies Inc.). Libraries were sequenced on a MiSeq instrument (Illumina) using v2 reagents with 2 by 250-bp paired-end reads. For Oxford Nanopore (ONT), genomic libraries were prepared using a ligation sequencing kit (SQK-LSK109) strictly according to the ONT protocol “Genomic DNA by ligation (SQK-LSK109)” (GDE_0963_v109_revS_14Aug2019) with recommended third-party consumables, i.e., NEBNext formalin-fixed, paraffin-embedded (FFPE) repair mix and NEBNExt end repair/dA-tailing module (New England BioLabs, Ipswich, MA, USA). Then, the libraries were sequenced with the MinION Mk 1B for 48 h using flow cell FC-106D-R9.4.1. ONT base calling was performed with ONT Albacore sequencing pipeline software (ver. 1.2.6).

### Genome assembly.

Unicycler (ver. 0.4.0) software with default options under the normal mode was used for hybrid genome assembly and circularization of Illumina and ONT reads. The assembly was preceded by cleaning the Illumina and ONT reads from adapters using, Trimmomatic (ver. 0.36) (parameters: PE -phred33 ILLUMINACLIP: NexteraPE-PE.fa:2:30:10 LEADING:3 TRAILING:3 SLIDINGWINDOW:4:15 MINLEN:36) (55) and Porechop (ver 0.2.0, https://github.com/rrwick/Porechop) software, respectively, using the program default parameters. In addition, the Filtlong tool (ver. 0.1.1, https://github.com/rrwick/Filtlong) was used for filtering ONT reads by quality and selecting ≥1,000-bp reads for the assembly (parameters: min_length, 1000; keep_percent, 90; target_bases, 500,000,000; trim-split, 100; mean_q_weightm 10).

### Pan-genome and pan-plasmidome assignation.

The B. mycoides pan-genome size and the gene categories were obtained with Roary software ver. 3.13.0, applying default options except identity for the blastp algorithm, which was set to 90% instead of 95% (56). Genes were divided into four categories as recommended by Inglin et al. (34) and Kaas et al. (57), e.g., core genes (common to at least 99% of strains), soft-core genes (present in between 95% and 99% of strains), shell genes (present in between 15% and 94% of strains), and cloud genes (present in less than 15% of strains). The Roary software ver. 3.13.0 with default options was also used in the B. mycoides pan-plasmidome determination, considering all plasmid genes present in the analyzed isolates and dividing them into four pan-plasmidome categories by extending the concept of the pan-genome (for details, see Table 1). Pan-genomes and pan-plasmidomes were computed for all B. mycoides isolates under study jointly (*n* = 35) and separately for the BPN (*n* = 21) and JAS (*n* = 14) bacilli. The pan-plasmidome was considered a subset of the B. mycoides pan-genome.

Similarity of plasmids was assessed based on DNA 30-kb synteny fragments using the Sibelia program ver. 3.0.7 (58). The B. mycoides nucleotide sequence-based plasmid similarity network was constructed with the plasmid ATLAS (pATLAS) software (http://www.patlas.site) using the program default options and databases (59).

Antibiotic resistance genes and virulence determinants were searched using ABRicate v0.8 (https://github.com/tseemann/abricate), with the ResFinder, CARD ver. 2.0.3, and VFDB databases. Prophages present in B. mycoides under study were indicated with the use of PHASTER web server (accessed June 2020) (60), while insertion sequences were determined using ISEScan ver. 1.7.1 (61).

### Phylogenetic analysis.

The phylogenetic analysis was prepared with the use of nucleotide sequences of B. mycoides isolates under study. Furthermore, the nucleotide sequences of B. mycoides KBAB4, B. mycoides DSM 11821, B. mycoides ATCC 6462 (the species type strain), B. cereus ATCC 14589, B. cereus ATCC 10987, B. thuringiensis ATCC 10792, B. thuringiensis HD1, B. anthracis Ames, B. wiedmannii FSL W8-0169, B. toyonensis BCT-7112, B. cytotoxicus NVH391098, and B. subtilis 168 were included.

Construction of the tree reflecting the phylogenetic relationship of B. mycoides isolates was achieved using Reference Sequence Alignment-Based Phylogeny (RealPhy) program ver. 112 (62) with default options and RAxML ver. 8.2.10 software as the tree building tool with a bootstrap of 1,000 replicates (parameters: -m GTRGAMMA -p 12345 -s polymorphisms_move.phy -o B. subtilis strain 168 -N 1000). The gene content similarity between strains was assessed using an average nucleotide identity (ANI) analysis that generated a gene content correlation matrix visualized as a heat map.

The distance between the core and accessory genes of pairs of the isolates under study was achieved with Population Partitioning Using Nucleotide *k*-mers (PopPUNK) program (https://web.poppunk.net/) based on *k*-mer differences (63). Default options were applied in all analyses.

### Gene functional annotation.

Genes of all B. mycoides strains under study were assigned to Kyoto Encyclopedia of Genes and Genomes (KEGG) functional categories using eggnog-mapper (ver. 5.0) software (64) along with the BLAST of KEGG Orthology And Links Annotation (BlastKOALA) web server (65). In addition, the enrichM tool (https://github.com/geronimp/enrichM) was applied to compare differences in occurrence of KEGG categories between B. mycoides from the Białowieża National Park and from the Jasienówka farm using the Fisher exact test and principal-component analysis (PCA). The differences were interpreted as statistically significant at a *P* value of <0.05.

### Plasmid isolation.

To verify the *in silico* analyses, plasmids of up to 100 kb in B. mycoides isolates were purified using the HiSpeed plasmid maxi kit (Qiagen GmbH, Hilden, Germany) according to the manufacturer’s protocol, and separated in 1% Prona Plus agarose gel (Laboratories Conda, Spain) as described by Swiecicka et al. (8). Following the visualization with a Midori stain, the plasmid sizes were assessed by comparison with those in B. thuringiensis strain IS5056 (66).

### Data availability.

The data sets supporting this study have been deposited in the National Center for Biotechnology Information (NCBI) database under accession numbers: CP035953 to CP035961 (BPN03/1), CP035962 to CP035964 (BPN07/3), CP066847 to CP066850 (BPN08/1), CP035965 to CP035972 (BPN09/1), CP072065 to CP072068 (BPN29/1), CP035994 to CP035996 (BPN36/2), CP035997 to CP035998 (BPN36/3), CP035999 to CP036003 (BPN37/1), CP036004 to CP036008 (BPN37/2), CP036009 to CP036013 (BPN43/2), CP036017 to CP036022 (BPN51/1), CP036023 to CP036025 (BPN52/2), CP036026 to CP036033 (BPN54/2), CP036034 to CP036039 (BPN57/2), CP036042 to CP036045 (BPN58/4), CP035973 to CP035977 (BPN102), CP035978 to CP035983 (BPN121), CP035984 to CP035986 (BPN211), CP031071 to CP031077 (BPN401), CP036040 to CP036041 (BPN573), CP036046 to CP036051 (BPN601), CP072061 to CP072064 (JAS06/1), CP036064 to CP036069 (JAS06/3), CP072057 to CP072060 (JAS12/5), CP071811 to CP071818 (JAS15/1), CP036099 to CP036100 (JAS23/1), CP036137 to CP036144 (JAS83/3), CP072055 to CP072056 (JAS85/1), CP036145 to CP036148 (JAS95/4), CP071808 to CP071810 (JAS014), CP036102 to CP036108 (JAS391), CP036115 to CP036120 (JAS481), CP036121 to CP036131 (JAS635), CP036132 to CP036136 (JAS823), CP036074 to CP036084 (JAS1004). For details see, Table S1.
