# Supplementary material for: Pan-Genome Portrait of Bacillus mycoides Provides Insights into the Species Ecology and Evolution
Source: Microbiol Spectr. 2021 Jul 21;9(1):10.1128/spectrum.00311-21. doi: 10.1128/spectrum.00311-21 (PMC8552610; doi:10.1128/spectrum.00311-21)
Supplement: SUPPLEMENTAL FILE 1 — Supplemental material. Download SPECTRUM00311-21_Supp_1_seq12.pdf, PDF file, 1.8 MB. [file spectrum00311-21_supp_1_seq12.pdf]

SUPPLEMENTAL MATERIAL

Pan-genome portrait of *Bacillus mycoides* provides insights into the species ecology and evolution

Fiedoruk K., Drewnowska J.M., Mahillon J., Zambrzycka M., Swiecicka I

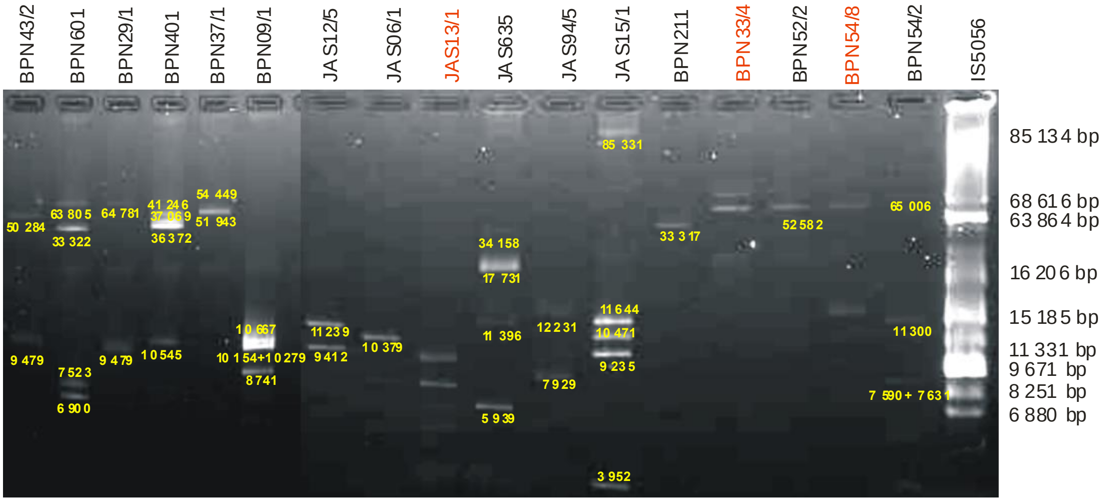

FIG S1 Plasmids in representatives of *Bacillus mycoides* used in the study.

Plasmids were purified using the HiSpeedPlasmid Maxi Kit (Qiagen GmbH, Hilden, Germany) following the manufacturer's protocol, and separated in 1% Prona Plus agarose gel (Laboratories Conda, Spain) as described in Swiecicka *et al.* [2008]. Following the visualization with Midori stain, the plasmid sizes were assessed by comparison with those in *B. thuringiensis* strain IS5056 [Murawska *et al.*, 2013]. Application of the HiSpeed Plasmid Maxi Kit (Qiagen GmbH) allows isolation of plasmids of size up to 100 kb. Paths indicated as BPN33/4, BPN54/8, and JAS13/1 picture plasmids of *Bacillus cereus sensu lato* not included in the study.

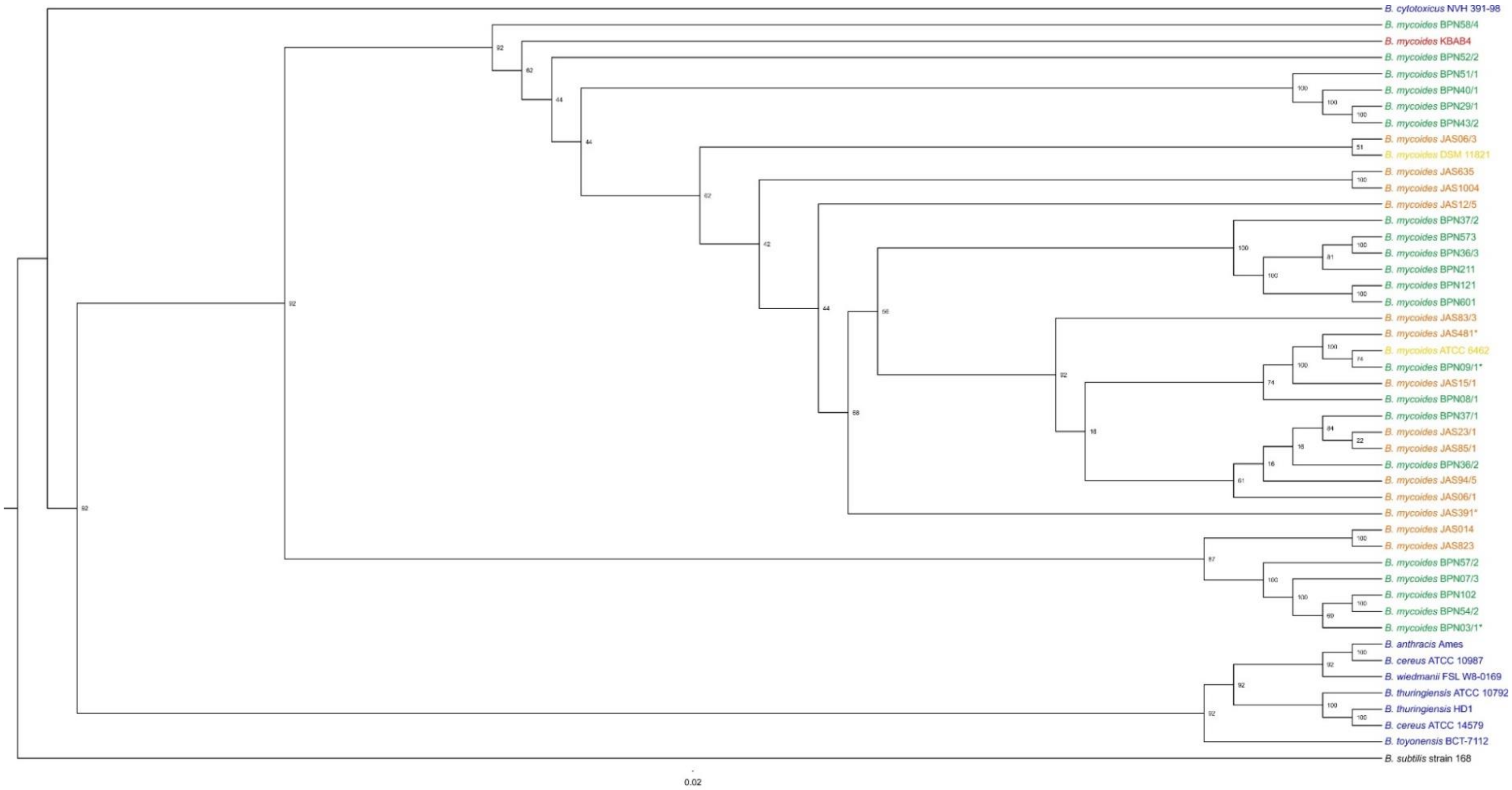

FIG S2 *Bacillus mycoides* phylogenetic tree.

The rooted tree was achieved based on aligned SNP positions in chromosomes identified using RealPhy software ver. 113 [Bertels *et al*, 2014] with default options and RAxML ver. 8.2.10 software as the tree building tool with bootstrap 1000 replicates (parameters: -m GTRGAMMA -p 12345 -s polymorphisms_move.phy -o *B. subtilis* strain 168 -N 1000). *B. subtilis* strain 168 (marked in black) was used as outgroup, while *B. mycoides* KBAB4 (marked in red) as a reference strain. Two additional reference *B. mycoides* strains (marked in yellow), ATCC 6462 (a type strain) and DSM 11821, as well as the remaining members of *B. cereus s. l.* species (marked in blue) were added for comparison. Isolates from Białowieża National Park are marked in green, while those from the Jasienówka farm in orange. Strains forming rhizoidal colonies on solid media are indicated with an asterisk. The node labels refer to bootstrap confidence values (a proportional branch transformation was performed using FigTree ver. 1.4 software for better clarity).

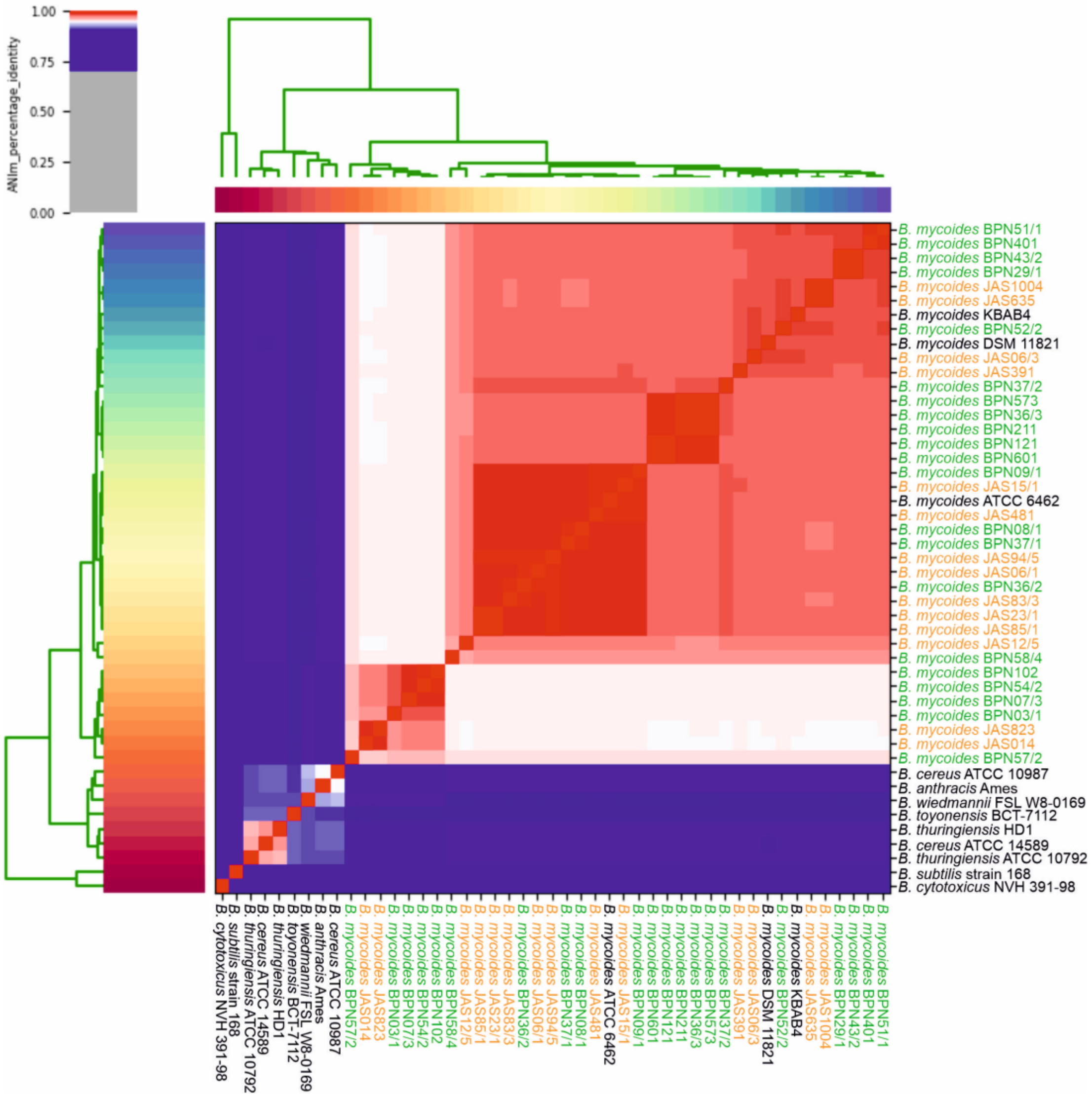

FIG S3 Heatmap and dendrogram representing average nucleotide identity (ANI) of the *B. mycoides* isolates under study (n=35; isolates from Białowieża National Park are marked in green, while those from the Jasienówka farm in yellow) and other *B. cereus s. l.* species (n=11) as well as *B. subtilis* str. 168 (n=1) (marked in black).

The calculation was performed using pyani script (https://github.com/widdowquinn/pyani) and MUMmer (NUCmer) aligner (pyani command syntax: average_nucleotide_identity.py -m ANIm).

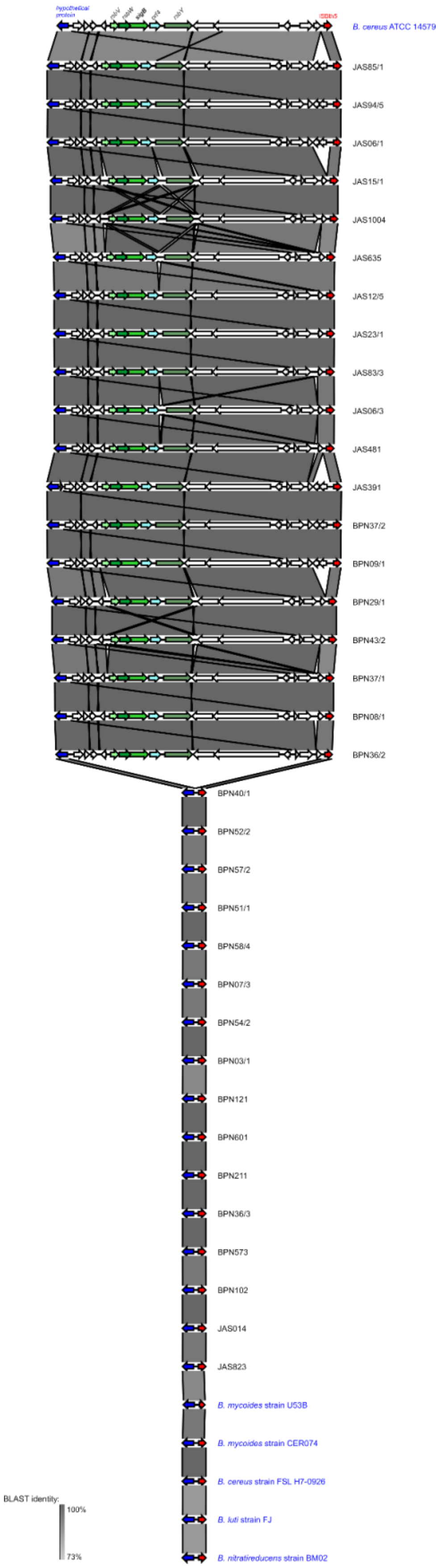

FIG S4 BLAST comparison of the chromosomal locus containing sigma B operon (*rsbV*, *rsbW*, *sigB*, *orf4* and *rsbY*) among *B. mycoides*.

The isolates under study (n=35) are shown in black. *B. cereus* strain ATCC 14579 [GenBank: AE016877.1], *B. cereus* strain FSL H7-0926 [GenBank: LOBD01000035.1], *B. mycoides* strain U53B [GenBank: JABURZ010000001.1], *B. mycoides* strain CER074 [GenBank: AHDT01000027.1], *B. luti* strain FJ [GenBank: CP040336.1], and *B. nitratireducens* strain BM02 [GenBank: CP047366.1] (shown in blue) were added as references. BLAST analysis was performed and visualized using Easyfig ver. 2.2.5 [Sullivan *et al*., 2011].

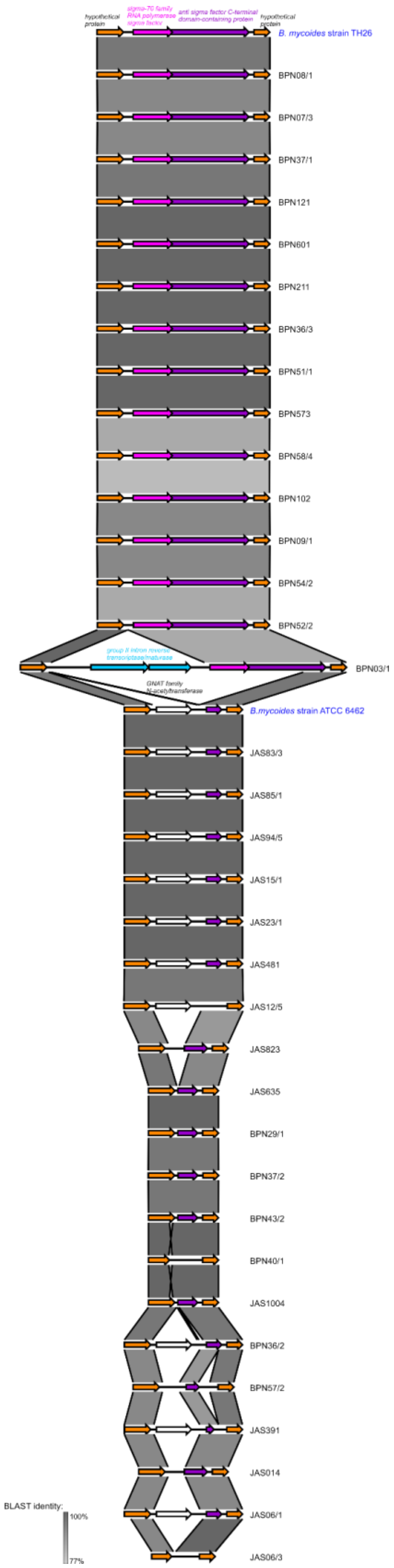

FIG S5 BLAST comparison of the chromosomal locus containing operon encoding sigma^{70} family RNA polymerase sigma factor (WP_061688722.1) and anti-sigma factor C-terminal domain-containing protein (WP_063218368.1) among *B. mycoides*

The isolates under study (n=35) are shown in black. *B. mycoides* strain ATCC 6462 [Genbank: CP009692] and *B. mycoides* strain TH26 [Genbank: CP037992] (shown in blue) were added as references. BLAST analysis was performed and visualized using Easyfig ver. 2.2.5 (Sullivan *et al*. 2011).

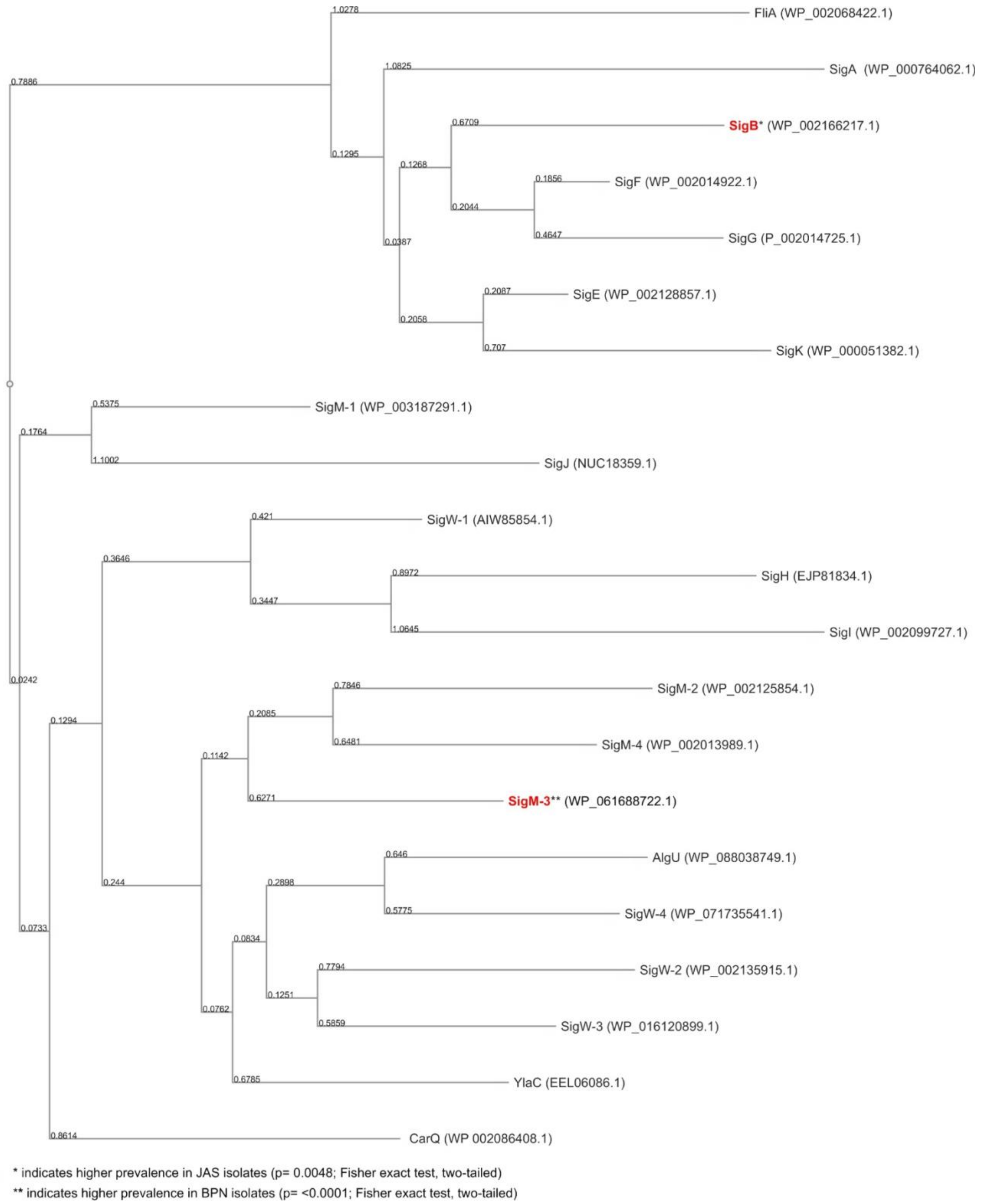

FIG S6 Neighbor-joining phylogenetic tree of sigma^{70} factors present in *B. mycoides* strains under study (BPN n=21; JAS n=14).

GenBank accession numbers of representative protein sequences are in brackets; red color indicates sigma^{70} factors significantly more prevalent in JAS (SigB) or BPN (SigM-3) isolates. MAFFT online service was used to align protein sequences (MAFFT L-INS-i strategy) and create dendrogram (a neighbor-joining method with a bootstrap value of 500 for the conserved sites - 81 amino acids) [Katoh *et al.* 2019].

Supplemental Material References

Bertels F, Silander OK, Pachkov M, Rainey PB, van Nimwegen E. 2014. Automated reconstruction of whole-genome phylogenies from short-sequence reads. Mol Biol Evol 31:1077–1088.

Carroll LM, Wiedmann M, Kovac J. 2020. Proposal of a taxonomic nomenclature for the *Bacillus cereus* group which reconciles genomic definitions of bacterial species with clinical and industrial phenotypes. mBio 11:e00034-20.

Drewnowska JM, Swiecicka I. 2013. Eco-genetic structure of *Bacillus cereus sensu lato* populations from different environments in Northeastern Poland. PloS One 8:e80175.

Katoh K, Rozewicki J, Yamada KD. 2019. MAFFT online service: multiple sequence alignment, interactive sequence choice and visualization. Brief Bioinform 20:1160–1166.

Lees JA, Harris SR, Tonkin-Hill G, Gladstone RA, Lo SW, Weiser JN, Corander J, Bentley SD, Croucher NJ, 2019. Fast and flexible bacterial genomic epidemiology with PopPUNK. Genome Res 29:304-316.

Murawska E, Fiedoruk K, Bideshi DK, Swiecicka I. 2013. Complete genome sequence of *Bacillus thuringiensis* subsp. *thuringiensis* IS50056, an isolate highly toxic to *Trichoplusia ni.* Genome Announc 21:e0010813.

Swiecicka I, Bideshi DK, Federici BA. 2008. Novel isolate of *Bacillus thuringiensis* subsp. *thuringiensis* that produces a quasicuboidal crystal of Cry1Ab21 toxic to larvae of *Trichoplusia ni*. Appl Environ Microbiol 74:923-930.

Sullivan MJ, Petty NK, Beatson SA. 2011. Easyfig: a genome comparison visualizer. Bioinformatics 27:1009–1010.

Table S1. *Bacillus mycoides* isolates from Northeastern Poland under study.

Bacillus mycoides isolate [a,b]	ST [c]	No. of contigs	Genome coverage			GenBank Accession Number
			Nanopore	Illumina	Summary	
BPN03/1*	711	9	155.188	67.422	222.61	CP035953-CP035961
BPN07/3	659	3	173.24	105.62	278.86	CP035962-CP035964
BPN08/1	649	4	186.44	49.83	236.27	CP066847-CP066850
BPN09/1*	625	8	21.903	62.464	84.37	CP035965-CP035972
BPN29/1	678	4	97.768	29.705	127.47	CP072065-CP072068
BPN36/2	650	3	114.213	54.511	168.72	CP035994-CP035996
BPN36/3	742	2	61.736	50.491	112.23	CP035997-CP035998
BPN37/1	655	5	27.873	80.611	108.48	CP035999-CP036003
BPN37/2	662	5	70.775	30.392	101.167	CP036004-CP036008
BPN43/2	678	5	26.337	48.04	74.38	CP036009-CP036013
BPN51/1	667	6	191.833	94.72	286.55	CP036017-CP036022
BPN52/2	657	3	35.966	156.588	192.55	CP036023-CP036025
BPN54/2	668	8	103.726	70.901	174.63	CP036026-CP036033
BPN57/2	739	6	194.444	55.885	250.329	CP036034-CP036039
BPN58/4	708	4	92.789	127.311	220.1	CP036042-CP036045
BPN102	1507	5	94.29	54.198	148.49	CP035973-CP035977
BPN121	1519	6	51.143	89.154	140.3	CP035978-CP035983
BPN211	742	3	33.78	138.54	172.32	CP035984-CP035986
BPN401	1568	7	155.053	42.421	197.47	CP031071-CP031077
BPN573	742	2	97.751	54.327	152.08	CP036040-CP036041
BPN601	1519	6	33.865	88.915	122.78	CP036046-CP036051
JAS06/1	222	4	25.325	110.22	135.55	CP072061-CP072064
JAS06/3	617	6	57.109	76.597	133.71	CP036064-CP036069
JAS12/5	618	4	30.454	70.06	100.51	CP072057-CP072060
JAS15/1	624	8	120.689	53.975	174.664	CP071811-CP071818
JAS23/1	727	2	44.893	86.151	131.04	CP036099-CP036100
JAS83/3	615	4	155.172	40.862	196.034	CP036137-CP036144
JAS85/1	410	2	71.677	75.771	147.45	CP072055-CP072056
JAS94/5	726	4	122.613	73.317	195.93	CP036145-CP036148
JAS014	1511	3	86.944	53.514	140.46	CP071808-CP071810
JAS391*	671	7	156.316	36.63	192.95	CP036102-CP036108
JAS481*	624	6	102.011	85.849	187.86	CP036115-CP036120
JAS635	695	11	89.614	78.698	168.31	CP036121-CP036131
JAS823	1514	5	101.123	74.148	175.27	CP036132-CP036136
JAS1004	695	11	119.621	39.97	159.59	CP036074-CP036084

[a] BNP, isolates from soil samples collected in Białowieża National Park, (N 52°42′, E 23°54′); JAS, isolates from soil samples picked up in a farmland in Jasienowka (N 52°30′, E 22°58′); Northeastern Poland. For details see Drewnowska and Swiecicka [2013];

[b] an isolate forming rhizoidal colonies on agar plates is indicated with an asterisk;

[c] ST, sequence type determined with the use of Multilocus Sequence Types (MLST). For details see Drewnowska and Swiecicka [2013].

Table S2. Genome characteristics of *Bacillus mycoides* isolates under study.

Bacillus mycoides isolate	ST	Contig	Length [bp]	GC content [%]	Number of CDSs	Number of rRNA	tRNA	tmRNA	Genome length [bp]	Total length of plasmids [bp]	Portion of plasmids in a genome [%]	PopPUNK cluster
		c1	5 010 935	35.7	5 037	43	107	1				
		c2	321 896	34.1	300							
		c3	210 021	33.1	225							
		c4	15 401	32.7	13							
BPN3/1*	711	c5	11 177	32.4	13				5 602 899	591 964	10.57	I
		c6	10 858	35.7	11							
		c7	9 615	34.1	10							
		c8	9 219	33.1	9							
		c9	3 777	32.7	4							
		c1	5 145 716	32.4	5 124	43	108	1				
BPN07/3	659	c2	361 734	32.2	359				5 740 114	594 398	10.36	I
		c3	232 664	31.9	216							
		c1	5 281 449	32.8	5 740	42	106	1				
BPN08/1	649	c2	523 425	35.1	535				6 116 513	835 064	13.65	II
		c3	228 644	35.7	257							
		c4	82 995	33.0	94							
		c1	5 340 691	33.6	5 357	42	107	1				
		c2	397 298	35.5	386							
		c3	249 030	33.4	253							
BPN09/1*	625	c4	107 498	33.4	105				6 134 358	793 667	12.94	II
		c5	10 667	32.5	13							
		c6	10 279	28.8	12							
		c7	10 154	32.5	9							
		c8	8 741	32.8	12							
		c1	5 243 038	35.6	5 263	42	107	1				
BPN29/1	678	c2	400 631	33.6	372				5 717 929	474 891	8.31	II
		c3	64 781	33.1	81							
		c4	9 479	32.0	7							
		c1	5 347 123	35.5	5 282	42	107	1				
BPN36/2	650	c2	538 903	33.2	480				5 886 026	538 903	9.16	II
		c3	14 792	36.7	26							
BPN36/3	742	c1	5 281 953	35.5	5 277	42	107	1	5 763 140	481 187	8.35	II
		c2	481 187	33.6	412							
		c1	5 322 329	35.5	5 360	42	107	1				
		c2	374 906	34.1	332		1					
BPN37/1	655	c3	225 867	33.4	232				6 029 494	707 165	11.73	II
		c4	54 449	36.8	76							
		c5	51 943	35.6	72							
		c1	5 490 503	35.5	5 512	42	106	1				
		c2	371 005	33.3	329							
BPN37/2	662	c3	77 113	34.5	97				5 956 071	465 568	7.81	II
		c4	9 322	30.2	9							
		c5	8 128	29.1	10							
		c1	5 369 163	35.6	5 293	42	107	1				
		c2	394 614	33.7	341							
		c3	50 441	37.0	76							
BPN401	1568	c4	41 246	34.8	59				5 939 450	570 287	9.6	II
		c5	37 069	30.2	40							
		c6	36 372	35.2	44							
		c7	10 545	29.3	12							
		c1	5 205 885	35.6	5 184	42	107	1				
		c2	400 360	33.6	368							
BPN43/2	678	c3	75 408	31.7	105				5 741 417	535 532	9.33	II
		c4	50 285	32.4	59							
		c5	9 479	32.0	7							
		c1	5 281 951	35.6	5 256	42	107	1				
		c2	318 875	33.8	275							
BPN51/1	667	c3	123 831	33.0	132				5 779 348	497 397	8.61	II
		c4	37 113	30.9	35		1					
		c5	9 651	31.6	9							
		c6	7 927	32.2	10							
		c1	5 266 779	35.6	5 236	42	107	1				
BPN52/2	657	c2	479 471	33.3	421		1		5 798 832	532 053	9.18	II
		c3	52 582	36.6	84							
		c1	5 208 286	35.7	5 234	43	108	1				
		c2	419 002	32.9	401		1					
		c3	232 193	33.5	216							
BPN54/2	668	c4	65 006	33.2	63				5 956 428	748 142	12.56	I
		c5	11 300	30.3	13							
		c6	7 631	35.7	10							
		c7	7 590	36.8	10							
		c8	5 420	36.6	8							
		c1	5 067 631	35.6	5 003	43	109	1				

BPN57/2	739	c2	369 824	33.4	345				5 554 158	486 527	8.76	I
		c3	92 054	32.8	91							
		c4	8 597	34.6	9							
		c5	8 039	36.9	7							
		c6	8 013	32.7	8							
BPN58/4	708	c1	5 248 764	35.6	5 251	42	107	1	5 951 634	702 870	11.81	II
		c2	437 823	34.0	385							
		c3	211 646	32.4	171							
		c4	53 401	35.4	76							
BPN102	1507	c1	5 093 759	35.7	5 084	43	108	1	5 747 492	653 733	11.37	I
		c2	357 042	33.0	357							
		c3	231 973	33.5	212							
		c4	54 371	36.1	71							
		c5	10 347	30.7	10							
BPN121	1519	c1	5 243 491	35.6	5 244	42	107	1	5 751 434	507 943	8.83	II
		c2	396 505	33.6	319							
		c3	63 697	33.0	71							
		c4	33 318	33.4	40							
		c5	7 523	35.6	10							
		c6	6 900	33.0	9							
BPN211	742	c1	5 265 340	35.6	5 263	42	107	1	5 809 871	544 531	9.37	II
		c2	511 214	33.5	434							
		c3	33 317	33.4	41							
BPN573	742	c1	5 281 925	35.5	5 282	42	107	1	5 762 966	481 041	8.35	II
		c2	481 041	33.6	413							
BPN601	1519	c1	5 245 175	35.6	5 247	42	107	1	5 753 119	507 944	8.83	II
		c2	396 394	33.6	319							
		c3	63 805	33.0	71							
		c4	33 322	33.4	40							
		c5	7 523	35.6	9							
		c6	6 900	33.0	9							
JAS06/1	222	c1	5 352 400	35.5	5 371	43	107	1	5 938 126	585 726	9.86	II
		c2	487 361	33.0	438							
		c3	87 986	33.0	86							
		c4	10 379	30.8	11							
JAS06/3	617	c1	5 348 830	35.6	5 302	42	107	1	5 865 739	516 909	8.81	II
		c2	233 674	33.3	233							
		c3	207 206	33.4	204							
		c4	46 669	36.3	75							
		c5	19 880	31.9	17							
		c6	9 480	31.0	11							
JAS12/5	618	c1	5 265 397	35.6	5 256	42	107	1	5 491 933	226 536	4.12	II
		c2	205 885	33.4	184							
		c3	11 239	34.9	11							
		c4	9 412	29.8	11							
JAS15/1	624	c1	5 291 025	35.6	5 332	42	107	1	6 010 975	719 950	11.98	II
		c2	390 912	33.9	414							
		c3	208 405	32.5	213							
		c4	85 331	33.1	84							
		c5	11 644	31.3	13							
		c6	10 471	31.4	12							
		c7	9 235	31.6	10							
		c8	3 952	34.4	3							
JAS23/1	727	c1	5 313 017	35.5	5 303	42	107	1	5 821 834	508 817	8.74	II
		c2	508 817	33.1	458							
JAS83/3	615	c1	5 390 204	35.5	5 754	42	108	1	6 005 285	615 081	10.24	II
		c2	545 956	33.5	519		2					
		c3	62 102	43.7	88							
		c4	7 023	30.4	7							
JAS85/1	410	c1	5 339 421	35.5	5 350	43	108	1	5 821 266	481 845	8.28	II
		c2	481 845	32.9	431							
JAS94/5	726	c1	5 275 222	35.6	5 261	42	108	1	5 642 620	367 398	6.51	I
		c2	347 238	34.1	301							
		c3	12 231	28.4	13							
		c4	7 929	32.2	10							
JAS014	1511	c1	5 146 518	35.7	5 117	43	107	1	5 342 665	196 147	3.67	I
		c2	185 643	33.0	168							
		c3	10 504	32.5	11							
JAS391*	671	c1	5 235 951	35.6	5 228	42	108	1	6 056 335	820 384	13.55	II
		c2	405 965	34.2	383							
		c3	247 123	32.8	242							
		c4	109 920	33.7	112							
		c5	37 394	35.9	35							
		c6	10 082	31.2	11							
		c7	9 900	32.4	12							
JAS481*	624	c1	5 296 574	35.6	5 242	42	106	1	6 014 015	717 441	11.93	II
		c2	334 352	33.8	343							
		c3	237 265	32.7	240							
		c4	124 309	33.4	127							

		c5	11 818	31.3	14							
		c6	9 697	31.9	10							
		c1	5 473 370	35.6	5 514	42	109	1				
		c2	192 434	33.7	187							
		c3	139 054	34.1	143							
		c4	97 153	32.9	105							
		c5	73 718	34.5	83							
JAS635	695	c6	70 690	35.1	80				6 163 981	690 611	11.2	II
		c7	48 338	35.2	49							
		c8	34 158	35.0	38							
		c9	17 731	31.9	14							
		c10	11 396	30.7	13							
		c11	5 939	34.1	4							
		c1	5 114 971	35.6	5 097	43	107	1				
		c2	162 314	33.2	157							
JAS823	1514	c3	52 218	36.9	84				5 344 184	229 213	4.29	I
		c4	10 034	30.2	10							
		c5	4 647	35.1	3							
		c1	5 407 642	35.6	5 432	42	109	1				
		c2	506 603	33.5	465							
		c3	198 488	33.7	205							
		c4	77 756	34.2	86							
		c5	48 337	35.2	50							
JAS1004	695	c6	36 057	35.3	39				6 338 271	930 629	14.68	II
		c7	16 651	32.8	16							
		c8	13 386	33.2	16							
		c9	12 044	32.4	10							
		c10	11 669	30.6	13							
		c11	9 638	29.7	10							

Legend:

***Bacillus mycodes* isolate,** isolates from soil samples collected in Białowieża National Park are indicated as BPN while these from soil samples picked up in a farmland in Jasienowka as JAS; isolates forming rhizoidal colonies on agar plates are marked with an asterisk;

ST, sequence type determined with the use of Multilocus Sequence Types (MLST) and available at the *B. cereus* group MLST database (https://pubmlst.org/organisms/bacillus-cereus). For details see Drewnowska and Swiecicka [2013];

Contig, in each isolate contig c1 indicates chromosome while others represent plasmids with an exception of c3 in BPN36/2 assigned to a phage;

Length [bp], a length of a contig in base pairs;

GC content [%], a percentage of GC nucleotides of a contig;

Number of CDSs, a number of coding DNA sequences in a contig;

Number of rRNA, tRNA, tmRNA, a number of DNA sequences encoding ribosomal RNA (rRNA), transfer RNA (tRNA), and transfer-messenger RNA (tmRNA). For clarity the lack of the RNAs genes on plasmids was not indicated.

Genome size [bp], a length of all contigs present in an isolate in base pairs;

Total length of plasmids [bp], a total length of all plasmids present in an isolate in base pairs;

Portion of plasmids in a genome [%], a percentage of the total sizes of all plasmids in a genome;

PopPUNK cluster, a cluster determined based on chromosomes distances achieved with PopPUNK [Lees *et. al.,* 2019].

Table S3. Summary of genome features of *Bacillus mycoides* under study.

B. mycoides [a]	Genome		Chromosome		Plasmid	
	**Average length [bp]**	**Range of length [bp]**	**Average length [bp]**	**Range of length [bp]**	**Range of No. per strain** [b]	**Range of length [bp]**
BPN (n=21)	5 833 689.8	5 554 158 – 6 134 358	5 249 613.6	5 010 935 – 5 490 503	1 - 8 (3.9)	3 777 – 538 903
JAS (n=14)	5 846 944.9	5 342 665 – 6 338 271	5 303 610.1	5 114 971 – 5 473 370	1 - 10 (4.5)	3 952 – 545 956

[a] BPN *B. mycoides* isolated from soil samples collected in Białowieża National Park; JAS *B. mycoides* isolated from soil samples picked up in a farm in Jasienowka Northeastern Poland (for details see Drewnowska and Swiecicka, 2013);
[b] average number of plasmids per strain is given in brackets.

Table S4. Prophages harboured by *Bacillus mycoides* isolates under study.

Bacillus mycoides isolate	ST	Prophage location	Phage length [kb]	Completeness	Score	Total No. of proteins encoded	Region position	Most common prophage	GC content [%]
BPN3/1*	711	chromosome (5 010 935 bp)	6.6	incomplete	10	0	1 988 928 - 1 995607	PHAGE_Bacill_phBC6A52_NC_004821(3)	34.54
		plasmid (321 896 bp)	25.8	incomplete	50	30	41 418 - 67 274	PHAGE_Bacill_BtCS33_NC_018085(4)	33.2
			43.7	intact	110	39	113 086 - 156 793	PHAGE_Escher_RCS47_NC_042128(2)	34.47
		plasmid (210 021 bp)	29.8	incomplete	40	22	76 643 - 106 472	PHAGE_Strept_Dp_1_NC_015274(3)	32.45
BPN07/3	659	chromosome (5 145 716 bp)	14.8	incomplete	50	11	1 061 021 - 1 075 873	PHAGE_Bacill_BtCS33_NC_018085(2)	34.91
			34.2	incomplete	20	24	1 991 383 - 2 025 631	PHAGE_Bacill_phBC6A52_NC_004821(4)	34.15
			19.1	incomplete	40	23	2 025 996 - 2 045 160	PHAGE_Bacill_vB_BhaS_171_NC_030904(8)	35.39
		plasmid (361 734 bp)	62.3	intact	140	47	49 418 - 111 805	PHAGE_Clostr_c_st_NC_007581(5)	31.25
BPN08/1	649	chromosome (4 369 454 bp)	45.2	questionable	70	30	2 196 513 - 2 241 804	PHAGE_Bacill_phBC6A52_NC_004821(3)	32.99
			22.7	incomplete	10	11	2 463 369 - 2 486 070	PHAGE_Bacill_phBC6A52_NC_004821(5)	33.94
		plasmid (523 425 bp)	23.4	incomplete	40	32	173 320 - 196 742	PHAGE_Bacill_phBC6A52_NC_004821(5)	34.61
			12.1	incomplete	10	21	461 530 - 473 693	PHAGE_Bacill_phBC6A51_NC_004820(3)	34.90
			11.6	incomplete	30	23	483 179 - 494 807	PHAGE_Bacill_IEBH_NC_011167(2)	33.08
BPN09/1*	625	chromosome (5 340 691 bp)	68.3	intact	100	95	2 273 987 - 2 342 381	PHAGE_Bacill_phBC6A51_NC_004820(50)	37.30
		plasmid (397 298 bp)	45.0	questionable	70	33	130 827 - 175 851	PHAGE_Bacill_IEBH_NC_011167(3)	33.57
			21.0	incomplete	20	23	247 633 - 268 656	PHAGE_Bacill_phBC6A52_NC_004821(4)	34.25
			44.0	intact	150	59	289 659 - 333 728	PHAGE_Bacill_IEBH_NC_011167(3)	33.33
		plasmid (249 030 bp)	32.2	questionable	70	26	2 669 - 34 926	PHAGE_Bacill_Gamma_NC_007458(4)	33.72
			31.7	questionable	70	21	129 407 - 161 162	PHAGE_Strept_Dp_1_NC_015274(3)	32.61
BPN29/1	678	chromosome (5 642 477 bp)	47.0	intact	140	58	390 375 - 437 441	PHAGE_Bacill_vB_BhaS_171_NC_030904(8)	35.67
			13.1	incomplete	20	18	4 345 565 - 4 358 689	PHAGE_Mycoba_Bxz1_NC_004687(1)	35.17
			10.1	incomplete	30	20	5 276 722 - 5 286 863	PHAGE_Bacill_BtCS33_NC_018085(3)	33.61
			29.9	incomplete	30	27	5 510 179 - 5 540 139	PHAGE_Bacill_phBC6A52_NC_004821(4)	31.26
BPN36/2	650	plasmid (538 903 bp)	14.5	incomplete	50	16	51 984 - 66 516	PHAGE_Escher_RCS47_NC_042128(2)	32.11
			35.6	incomplete	60	36	114 692 - 150 353	PHAGE_Escher_RCS47_NC_042128(2)	33.50
			31.1	incomplete	50	27	147 823 - 178 985	PHAGE_Bacill_BtCS33_NC_018085(4)	34.08
			7.7	incomplete	20	17	321 443 - 329 148	PHAGE_Bacill_BtCS33_NC_018085(2)	31.47
		plasmid (14 792 bp)	14.0	intact	122	24	282 - 14 378	PHAGE_Bacill_Wip1_NC_022094(22)	37.20
BPN36/3	742	chromosome (5 281 953 bp)	38.7	intact	110	38	670 138 - 708 892	PHAGE_Brevib_Jenst_NC_028805(7)	35.94
			19.3	incomplete	10	26	709 390 - 728 696	PHAGE_Bacill_phBC6A52_NC_004821(5)	33.13
			14.9	incomplete	40	22	4 910 912 - 4 925 902	PHAGE_Bacill_phi4J1_NC_029008(3)	34.53
		plasmid (481 187 bp)	28.5	incomplete	50	38	124 746 - 153 288	PHAGE_Bacill_IEBH_NC_011167(4)	34.52
			28.4	incomplete	40	31	200 871 - 229 338	PHAGE_Escher_RCS47_NC_042128(2)	33.92
			23.0	questionable	70	22	266 559 - 289 605	PHAGE_Clostr_c_st_NC_007581(6)	31.88
			27.9	incomplete	40	28	363 407 - 391 324	PHAGE_Escher_RCS47_NC_042128(2)	34.19
BPN37/1	655	chromosome (5 322 329 bp)	23.3	questionable	80	24	1 - 23 397	PHAGE_Bacill_WBeta_NC_007734(17)	36.31
			40.6	intact	110	54	4 518 948 - 4 559 567	PHAGE_Geobac_GBSV1_NC_008376(10)	34.82
			20.5	incomplete	10	28	5 298 347 - 5 318 897	PHAGE_Bacill_phBC6A52_NC_004821(10)	34.74
		plasmid (54 449 bp)	49.8	intact	120	76	1 - 49 877	PHAGE_Bacill_IEBH_NC_011167(24)	37.15
		plasmid (51 943 bp)	44	intact	120	61	7 243 - 51 339	PHAGE_Bacill_BMBtpLA_NC_028748(13)	35.14
BPN37/2	662	chromosome (5 490 503 bp)	30.8	incomplete	50	42	1 - 30 805	PHAGE_Bacill_phBC6A52_NC_004821(15)	35.19
			42.5	incomplete	20	28	1 306 984 - 1 349 538	PHAGE_Bacill_vB_BhaS_171_NC_030904(8)	35.19
			8.3	incomplete	50	10	1 573 734 - 1 582 099	PHAGE_Ralsto_RSA1_NC_009382(1)	36.10
			49.1	intact	130	63	2 411 797 - 2 460 967	PHAGE_Bacill_BtCS33_NC_018085(20)	34.61
			9.8	incomplete	30	17	5 480 589 - 5 490 430	PHAGE_Staphy_StB12_NC_020490(2)	34.16
		plasmid (371 005 bp)	51.9	questionable	80	44	223 913 - 275 814	PHAGE_Escher_RCS47_NC_042128(2)	32.48
		plasmid (77 113 bp)	31.2	intact	110	37	7 329 - 38 623	PHAGE_Bacill_1_NC_009737(4)	37.26
BPN43/2	678	chromosome (5 205 885 bp)	13.1	incomplete	20	18	2 307 742 - 2 320 866	PHAGE_Mycoba_Bxz1_NC_004687(1)	35.16
		plasmid (400 360 bp)	30.0	incomplete	40	28	199 133 - 229 170	PHAGE_Bacill_phBC6A52_NC_004821(3)	31.38
			10.1	incomplete	30	19	366 067 - 376 208	PHAGE_Bacill_BtCS33_NC_018085(3)	33.61
		plasmid (50 285 bp)	10.7	incomplete	50	15	1 -10 780	PHAGE_Bacill_1_NC_009737(3)	33.5
			18.3	incomplete	30	22	17 854 - 36 231	PHAGE_Bacill_BMBtpLA_NC_028748(4)	31.42
BPN51/1	667	chromosome (5 281 951 bp)	13.1	incomplete	20	18	2 313 165 - 2 326 295	PHAGE_Mycoba_Bxz1_NC_004687(1)	35.19
		plasmid (318 875 bp)	36.1	incomplete	60	40	219 202 - 255 312	PHAGE_Staphy_SPbeta_like_NC_029119(2)	33.04
BPN52/2	657	chromosome (5 266 779 bp)	30.1	incomplete	40	14	249 231 - 279 390	PHAGE_Bacill_G_NC_023719(2)	35.93
			45.9	intact	100	54	3 608 545 - 3 654 521	PHAGE_Bacill_vB_BhaS_171_NC_030904(8)	35.24
		plasmid (479 471 bp)	19.1	incomplete	50	27	315 683 - 334 874	PHAGE_Bacill_phBC6A52_NC_004821(2)	33.46
			11.6	incomplete	30	23	434 554 - 446 228	PHAGE_Bacill_BtCS33_NC_018085(2)	33.03
		phage (51 394 bp)	51.4	intact	110	83	1 - 51 394	PHAGE_Bacill_IEBH_NC_011167(16)	36.65
BPN54/2	668	chromosome (5 208 286 bp)	68.7	intact	130	65	442 024 - 510 745	PHAGE_Lister_2389_NC_003291(15)	35.11
			22.6	incomplete	50	13	1 108 996 - 1 131 627	PHAGE_Bacill_BtCS33_NC_018085(2)	35.66
			45.2	questionable	70	51	2 145 459 - 2 190 733	PHAGE_Bacill_phBC6A52_NC_004821(21)	34.62
			27.3	questionable	90	24	3 579 783 - 3 607 159	PHAGE_Bacill_vB_BhaS_171_NC_030904(7)	34.41
		plasmid (419 002 bp)	34.8	intact	100	31	52 507 - 87 351	PHAGE_Clostr_c_st_NC_007581(4)	32.55
			39.8	questionable	90	33	306 003 - 345 829	PHAGE_Staphy_SPbeta_like_NC_029119(3)	33.9
BPN57/2	739	chromosome (5 067 631 bp)	5.7	incomplete	30	10	1 493 573 - 1 499 318	PHAGE_Bacill_G_NC_023719(2)	35.97
		plasmid (369 824 bp)	17.3	incomplete	40	38	49 799 - 67 107	PHAGE_Bacill_phi4J1_NC_029008(4)	34.17
			20.2	incomplete	40	15	74 904 - 95 162	PHAGE_Bacill_phBC6A52_NC_004821(4)	33.2
BPN58/4	708	chromosome (5 248 764 bp)	56.7	intact	110	59	2 588 591 - 2 645 291	PHAGE_Bacill_phBC6A52_NC_004821(16)	34.36
			44.6	intact	120	48	3 606 224 - 3 650 881	PHAGE_Bacill_vB_BhaS_171_NC_030904(8	35.12
		plasmid 437 823 bp)	14.2	incomplete	20	22	61 165 - 75 433	PHAGE_Bacill_BMBtpLA_NC_028748(4)	33.55
			7.2	incomplete	40	12	218 612 - 225 828	PHAGE_Escher_RCS47_NC_042128(2)	32.38
		plasmid (53 401 bp)	38.9	questionable	90	64	4 150 - 43 064	PHAGE_Bacill_1_NC_009737(10)	36.55
BPN102	1507	plasmid (357 042 bp)	27.3	questionable	70	26	49 405 - 76 726	PHAGE_Clostr_c_st_NC_007581(4)	33.31
			23.9	incomplete	10	31	261 768 - 285 704	PHAGE_Escher_RCS47_NC_042128(2)	32.9
		plasmid (54 371 bp)	43.9	questionable	90	60	4 699 - 48 616	PHAGE_Bacill_IEBH_NC_011167(22)	36.89
BPN121	1519	chromosome (5 243 491 bp)	23.6	incomplete	20	40	2 049 533 - 2 073 151	PHAGE_Bacill_phBC6A52_NC_004821(6)	33.09
			20.2	incomplete	50	19	2 084 503 - 2 104 786	PHAGE_Brevib_Jenst_NC_028805(7)	35.98
			34.9	incomplete	60	44	3 466 843 - 3 501 766	PHAGE_Bacill_1_NC_009737(7)	35.60
			33.8	incomplete	40	25	3 497 115 - 3 530 914	PHAGE_Bacill_phi4B1_NC_028886(9)	33.03
		plasmid (396 505 bp)	28.0	incomplete	20	47	178 813 - 206 869	PHAGE_Staphy_SPbeta_like_NC_029119(2)	33.83
			26.0	questionable	80	23	229 539 - 255 608	PHAGE_Clostr_c_st_NC_007581(6)	31.62
			28.0	incomplete	40	28	338 377 - 366 438	PHAGE_Escher_RCS47_NC_042128(2)	34.27
		plasmid (33 318 bp)	19.9	incomplete	40	17	13 204 - 33 182	PHAGE_Bacill_BtCS33_NC_018085(4)	34.33
BPN211	742	chromosome (5 265 340 bp)	64.2	intact	110	81	2 060 575 - 2 124 814	PHAGE_Bacill_phBC6A52_NC_004821(8)	34.68
			14.9	incomplete	40	22	3 138 437 - 3 153 427	PHAGE_Bacill_phi4J1_NC_029008(3)	34.51
		plasmid (511 214 bp)	43.2	questionable	90	51	202 908 - 246 201	PHAGE_Bacill_phiCM3_NC_023599(3)	32.49
			27.6	incomplete	40	28	360 931 - 388 560	PHAGE_Escher_RCS47_NC_042128(2)	33.96
		plasmid (33 317 bp)	19.9	incomplete	40	18	13 204 - 33 181	PHAGE_Bacill_BtCS33_NC_018085(4)	34.35
BPN401	568	chromosome (5 369 163 bp)	13.1	incomplete	20	18	2 357 758 - 2 370 912	PHAGE_Mycoba_Bxz1_NC_004687(1)	35.2
			31.4	incomplete	40	26	2 361 789 - 2 393 226	PHAGE_Bacill_phBC6A52_NC_004821(5)	33.33
		plasmid (394 614 bp)	10.0	incomplete	30	16	51 921 - 62 006	PHAGE_Bacill_phi4B1_NC_028886(3)	34.01
			24.5	incomplete	40	29	67 465 - 92 032	PHAGE_Escher_RCS47_NC_042128(2)	33.18
			31.7	questionable	70	44	153 327 - 185 122	PHAGE_Bacill_phiCM3_NC_023599(3)	33.15
			27.2	questionable	80	28	207 791 - 235 006	PHAGE_Clostr_c_st_NC_007581(6)	31.6
			34.9	questionable	70	29	306 831 - 341 810	PHAGE_Bacter_Diva_NC_028788(2)	34.29
		plasmid (50 441 bp)	49.2	intact	100	77	1 - 49 208	PHAGE_Bacill_IEBH_NC_011167(33)	37.06
		plasmid (41 246 bp)	41.2	intact	110	59	1 - 41 206	PHAGE_Lister_2389_NC_003291(16)	34.84
		chromosome (5 281 925 bp)	19.3	incomplete	10	29	2 051 676 - 2 070 982	PHAGE_Bacill_phBC6A52_NC_004821(5)	33.13
			32.0	intact	110	37	2 071 342 - 2 103 430	PHAGE_Brevib_Jenst_NC_028805(7)	35.93
			14.9	incomplete	40	22	3 137 756 - 3 152 746	PHAGE_Bacill_phi4J1_NC_029008(3)	34.51

BPN573	742	plasmid (481 041 bp)	28.5	incomplete	50	38	124 682 - 153 224	PHAGE_Bacill_IEBH_NC_011167(4)	34.52
			28.4	incomplete	40	32	200 807 - 229 279	PHAGE_Escher_RCS47_NC_042128(2)	33.9
			23.0	questionable	70	22	266 500 - 289 546	PHAGE_Clostr_c_st_NC_007581(6)	31.88
			28.0	incomplete	40	28	363 351 - 391 412	PHAGE_Escher_RCS47_NC_042128(2)	34.28
BPN601	1519	chromosome (5 245 175 bp)	23.6	incomplete	20	40	2 049 235 - 2 072 855	PHAGE_Bacill_phBC6A52_NC_004821(6)	33.08
			20.2	incomplete	50	19	2 084 207 - 2 104 490	PHAGE_Brevib_Jenst_NC_028805(7)	35.98
			34.9	incomplete	60	44	3 466 952 - 3 501 875	PHAGE_Bacill_1_NC_009737(7)	35.6
			33.8	incomplete	40	25	3 497 224 - 3 531 023	PHAGE_Bacill_phi4B1_NC_028886(9)	33.03
		plasmid (396 394 bp)	28.0	incomplete	20	47	178 702 - 206 758	PHAGE_Staphy_SPbeta_like_NC_029119(2)	33.83
			26.0	questionable	80	23	229 428 - 255 497	PHAGE_Clostr_c_st_NC_007581(6)	31.62
			28.0	incomplete	40	28	338 266 - 366 327	PHAGE_Escher_RCS47_NC_042128(2)	34.27
		plasmid (33 322 bp)	19.9	incomplete	40	17	13 204 - 33 186	PHAGE_Bacill_BtCS33_NC_018085(4)	34.35
JAS06/1	222	chromosome (5 361 170 bp)	28.5	incomplete	30	45	1 798 951 - 1 827 469	PHAGE_Bacill_phBC6A52_NC_004821(6)	33.59
			19.7	incomplete	60	19	1 840 951 - 1 860 654	PHAGE_Brevib_Jenst_NC_028805(7)	36.4
			50.9	intact	120	60	2 681 449 - 2 732 353	PHAGE_Bacill_WBeta_NC_007734(15)	34.55
		plasmid (487 389 bp)	37.6	incomplete	60	34	263 545 - 301 243	PHAGE_Escher_RCS47_NC_042128(2)	33.6
			31.4	incomplete	50	16	289 548 - 321 019	PHAGE_Bacill_BtCS33_NC_018085(4)	34.9
			9.4	incomplete	40	14	439 612 - 449 098	PHAGE_Bacill_BtCS33_NC_018085(2)	32.68
JAS06/3	617	chromosome (5 348 830 bp)	70.6	intact	140	67	1 591 032 - 1 661 652	PHAGE_Bacill_phiCM3_NC_023599(30)	35.52
		plasmid (233 674 bp)	41.9	intact	150	55	2 411 - 44 397	PHAGE_Bacill_phIS3501_NC_019502(4)	32.86
			12.2	incomplete	50	21	132 805 - 145 032	PHAGE_Escher_RCS47_NC_042128(2)	33.89
			22.5	incomplete	60	31	208 219 - 230 798	PHAGE_Bacill_phi4J1_NC_029008(3)	33.95
		plasmid (46 669 bp)	45.5	questionable	70	70	774 - 46 317	PHAGE_Bacill_BMBtpLA_NC_028748(26)	36.29
JAS12/5	618	chromosome (5 233 313 bp)	12.1	incomplete	20	17	4 332 400 - 4 344 598	PHAGE_Shigel_SfIV_NC_022749(1)	33.84
JAS15/1	624	chromosome (5 291 025 bp)	70.1	intact	106	96	2 260 280 - 2 330 467	PHAGE_Bacill_phBC6A51_NC_004820(53)	37.19
		plasmid (390 912 bp)	41.1	questionable	90	39	188 790 - 229 946	PHAGE_Bacill_IEBH_NC_011167(4)	32.83
			26.5	questionable	70	43	235 935 - 262 528	PHAGE_Bacill_phBC6A52_NC_004821(4)	33.59
			44.8	questionable	70	37	316 882 - 361 719	PHAGE_Escher_RCS47_NC_042128(2)	33.53
		plasmid (536 117 bp)	36.4	questionable	80	22	95 977 - 132 388	PHAGE_Strept_Dp_1_NC_015274(3)	32.62
JAS23/1	727	plasmid (508 817 bp)	37.7	incomplete	60	36	283 108 - 320 854	PHAGE_Escher_RCS47_NC_042128(2)	33.65
			31.4	incomplete	60	29	309 159 - 340 630	PHAGE_Bacill_PfEFR_5_NC_031055(4)	34.89
			9.4	incomplete	40	14	461 045 - 470 531	PHAGE_Bacill_BtCS33_NC_018085(2)	32.68
JAS83/3	615	chromosome (5 390 204 bp)	45.5	incomplete	60	59	2 691 039 - 2 736 611	PHAGE_Bacill_PfEFR_5_NC_031055(22)	34.72
			59.7	intact	150	68	3 176 711 - 3 236 416	PHAGE_Bacill_WBeta_NC_007734(26)	35.17
		plasmid (545 956 bp)	25.0	intact	130	26	113 736 - 138 773	PHAGE_Clostr_c_st_NC_007581(4)	33.17
			21.8	incomplete	20	29	324 483 - 346 348	PHAGE_Bacill_phBC6A52_NC_004821(9)	33.68
			26.0	questionable	90	31	432 059 - 458 100	PHAGE_Sodali_phiSG1_NC_007902(2)	35.13
		plasmid (62 102 bp)	62.0	questionable	90	96	1 - 62-091	PHAGE_Paenib_Tripp_NC_028930(25)	43.67
JAS85/1	410	plasmid (482 275 bp)	38.2	incomplete	60	35	253 882 - 292 132	PHAGE_Escher_RCS47_NC_042128(2)	33.66
			31.4	incomplete	50	16	280 437 - 311 908	PHAGE_Bacill_BtCS33_NC_018085(4)	34.89
			9.4	incomplete	40	14	435 994 - 445 480	PHAGE_Bacill_BtCS33_NC_018085(2)	32.67
JAS94/5	726	plasmid (347 238 bp)	13.8	incomplete	30	17	47 700 - 61 539	PHAGE_Escher_RCS47_NC_042128(2)	33.85
JAS014	1511	chromosome (5 146 518 bp)	10.1	incomplete	10	11	16 650 - 26 808	PHAGE_Bacill_G_NC_023719(2)	37.97
			8.4	incomplete	30	10	258 261 - 266 737	PHAGE_Bacill_G_NC_023719(2)	37.16
			8.3	incomplete	10	9	312 698 - 321 044	PHAGE_Synech_ACG_2014e_NC_026928(2)	38.62
			8.0	incomplete	20	8	649 758 - 657 853	PHAGE_Plankt_PaV_LD_NC_016564(1)	38.15
			10.7	incomplete	20	12	786 373 - 797 150	PHAGE_Bacill_BtCS33_NC_018085(3)	34.83
			10.7	incomplete	10	12	1 185 434 - 1 196 178	PHAGE_Sphing_PAU_NC_019521(4)	36.71
			8.7	incomplete	20	12	1 771 487 - 1 780 264	PHAGE_Bacill_WBeta_NC_007734(2)	34.34
			12.5	incomplete	10	11	2 183 474 - 2 196 050	PHAGE_Thermu_OH2_NC_021784(1)	33.79
		plasmid (185 643 bp)	11.6	incomplete	20	13	909 - 12 584	PHAGE_Bacill_phBC6A51_NC_004820(3)	32.69
JAS391*	671	chromosome (5 235 951 bp)	41.4	incomplete	60	50	3 598 398 - 3 639 879	PHAGE_Bacill_phBC6A52_NC_004821(21)	34.7
		plasmid (405 965 bp)	37.8	incomplete	50	34	60 250 - 98 094	PHAGE_Escher_RCS47_NC_042128(2)	35.7
			31.7	incomplete	60	22	295 768 - 327 485	PHAGE_Staphy_SPbeta_like_NC_029119(2)	34.53
		plasmid (247 123 bp)	39.4	questionable	80	33	66 403 - 105 822	PHAGE_Staphy_SPbeta_like_NC_029119(2)	31.79
			36.5	questionable	80	32	112 425 - 149 018	PHAGE_Strept_Dp_1_NC_015274(3)	32.66
JAS481*	624	plasmid (334 352 bp)	8.9	incomplete	30	14	28 488 - 37 421	PHAGE_Bacill_BtCS33_NC_018085(4)	34.2
			35.6	intact	100	45	142 860 - 178 528	PHAGE_Bacill_phBC6A52_NC_004821(5)	34.16
			49.3	intact	140	61	189 062 - 238 399	PHAGE_Bacill_IEBH_NC_011167(4)	33.68
			22.9	incomplete	40	31	273 105 - 296 046	PHAGE_Escher_RCS47_NC_042128(2)	31.98
		plasmid (237 265 bp)	25.4	questionable	70	26	2 730 - 28 223	PHAGE_Bacill_Gamma_NC_007458(4)	32.89
			20.9	incomplete	60	21	113 687 - 134 673	PHAGE_Strept_Dp_1_NC_015274(3)	31.78
JAS635	695	chromosome (5 473 370 bp)	22.1	questionable	90	21	1 - 22 140	PHAGE_Bacill_WBeta_NC_007734(13)	36.3
			44.2	intact	150	63	931 844 - 976 126	PHAGE_Bacill_phIS3501_NC_019502(16)	36,00
			20.1	questionable	70	19	3 404 104 - 3 424 295	PHAGE_Bacill_vB_BhaS_171_NC_030904(7)	36.39
			30.3	incomplete	20	30	3 426 986 - 3 457 296	PHAGE_Bacill_phBC6A52_NC_004821(5)	35.55
			53.9	intact	130	74	3 573 293 - 3 627 255	PHAGE_Bacill_BtCS33_NC_018085(21)	35.23
			28.4	incomplete	40	50	5 444 912 - 5 473 369	PHAGE_Bacill_phBC6A52_NC_004821(11)	34.22
		plasmid (192 434 bp)	44.5	intact	130	45	11 089 - 55 683	PHAGE_Staphy_SPbeta_like_NC_029119(2)	33.22
			34.5	questionable	90	42	157 798 - 192 313	PHAGE_Bacill_phi4J1_NC_029008(3)	34.17
JAS823	1514	chromosome (5 114 971 bp)	9.7	incomplete	10	7	2 057 233 - 2 066 996	PHAGE_Bacill_phBC6A52_NC_004821(4)	34.47
		plasmid (162 314 bp)	20.6	incomplete	30	21	2 171 - 22 856	PHAGE_Bacill_phBC6A51_NC_004820(3)	33.49
		plasmid (52 218 bp)	51.3	intact	110	85	1 - 51 388	PHAGE_Bacill_IEBH_NC_011167(39)	36.95
JAS1004	695	chromosome (5 407 642 bp)	51.1	intact	100	64	355 682 - 406 787	PHAGE_Bacill_phBC6A52_NC_004821(14)	35.13
			44.2	intact	150	63	1 290 975 - 1 335 257	PHAGE_Bacill_phIS3501_NC_019502(16)	36.01
			53.9	intact	130	74	3 892 629 - 3 946 592	PHAGE_Bacill_BtCS33_NC_018085(21)	35.23
		plasmid (506 603 bp)	9.7	incomplete	40	18	472 790 - 482 537	PHAGE_Clostr_c_st_NC_007581(3)	33.81
		plasmid (198 488 bp)	30.9	questionable	70	37	2 519 - 33 419	PHAGE_Bacill_phBC6A51_NC_004820(4)	32.75
			25.9	questionable	90	24	171 668 - 197 567	PHAGE_Bacill_phi4J1_NC_029008(3)	36.44

Legend:

***Bacillus mycodes* isolate,** isolates from soil samples collected in Białowieża National Park are indicated as BPN while from soil samples picked up in a farmland in Jasienowka are marked as JAS; isolates forming rhizoidal colonies on agar plates are indicated with an asterisk;

ST, sequence type determined with the use of Multilocus Sequence Types (MLST); for details see Drewnowska and Swiecicka [2013] and https://pubmlst.org/organisms/bacillus-cereus;

Prophage location, a contig (a chromsome or a plasmid) containing a phage; a length of a contig is specified in parentheses;

Region length, a length of DNA identified as a phage in a contig;

Completeness, a prediction of whether the region contains a prophage achieved in the PHASTER software (with default options) based on a completeness score as follows: an intact phage, score >90%; an incomplete phage, score 70-90%; a questionable phage, score <70%;

Total No. of proteins encoded, a number of proteins encoded by a phage;

Region position, a start and an end of a phage on a bacterial chromosome or a plasmid;

Most common phage, a phage with the highest number of proteins most similar to those in the region;

GC content [%], a percentage of GC nucleotides of the region.

Table S5. Insertion sequences (ISs) in the *Bacilus mycoides* isolates under study.

Bacillus mycoides isolate	ST	IS location	IS family	No. of ISs	Total length of ISs [bp]	Part of a replicon [%]
BPN03/1*	711	chromosome (5 010 935 bp)	IS*200*/IS*605*	1	1 123	0.02
			IS*21*	2	2 67	0.05
			IS*3*	34	46 487	0.93
			IS*4*	6	6 928	0.14
			IS*6*	5	4 279	0.09
			IS*607*	4	6 213	0.12
			IS*66*	1	2 02	0.04
		plasmid (321 896 bp)	IS*110*	1	2 673	0.83
			IS*200*/IS*605*	12	13 842	4.3
			IS*3*	5	7 105	2.21
			IS*4*	6	7 158	2.22
			IS*6*	19	22 136	6.88
		plasmid (210 021 bp)	IS*200*/IS*605*	3	4 229	2.01
			IS*3*	3	3 94	1.88
			IS*4*	6	6 988	3.33
			IS*6*	11	9 828	4.68
		**Genome (5 602 899 bp)**	**Total**	**119**	**147 619**	**2.63**
BPN07/3	659	chromosome (5 145 716 bp)	IS*200*/IS*605*	5	7 749	0.15
			IS*21*	2	2 825	0.05
			IS*3*	9	10 095	0.2
			IS*4*	4	4 761	0.09
			IS*6*	5	5 807	0.11
			IS*607*	5	8 409	0.16
		plasmid (361 734 bp)	IS*200*/IS*605*	6	8 013	2.22
			IS*4*	8	12 576	3.48
			IS*6*	16	16 457	4.55
			new	1	1 371	0.38
		plasmid (232 664 bp)	IS*200*/IS*605*	5	8 12	3.49
			IS*3*	1	1 448	0.62
			IS*4*	12	1 7783	7.64
			IS*6*	9	7 335	3.15
		**Genome (5 740 114 bp)**	**Total**	**88**	**112 749**	**1.96**
BPN08/1	649	chromosome (4 369 454 bp)	IS*110*	2	3 21	0.07
			IS*21*	1	1 35	0.03
			IS*3*	1	3 069	0.07
			IS*4*	2	2 778	0.06
			IS*607*	5	8 519	0.19
		plasmid (782 170 bp)	IS*200*/IS*605*	1	1 305	0.17
			IS*21*	1	2 124	0.27
		plasmid (523 425 bp)	IS*200*/IS*605*	10	13 905	2.66
			IS*4*	13	13 217	2.53
			IS*6*	9	9 334	1.78
			new	1	1 743	0.33
		plasmid (228 664 bp)	IS*4*	2	3 586	1.57
			IS*5*	2	1 598	0.7
			IS*6*	2	2 256	0.99
		plasmid (82 995 bp)	IS*110*	1	3 062	3.69
		**Genome (6 116 513 bp)**	**Total**	**53**	**71 056**	**1.17**
BPN09/1*	625	chromosome (5 340 691 bp)	IS*200*/IS*605*	5	2 164	0.04
			IS*21*	2	2 826	0.05
			IS3	6	8 833	0.17
			IS*4*	6	5 314	0.1
			IS*607*	5	8 268	0.15
		plasmid (397 298 bp)	IS*110*	2	6 449	1.62
			IS*200*/IS*605*	8	9 969	2.51
			IS*3*	5	7 976	2.01
			IS*4*	5	4 725	1.19
			IS*6*	19	17 288	4.35
			IS256	1	917	0.37
			IS*3*	2	2 363	0.95

		plasmid (249 030 bp)	IS*4*	14	21 897	8.79
			IS*6*	10	8 701	3.49
			IS*66*	1	2 007	0.81
			new	1	4 445	1.78
		plasmid (107 498 bp)	IS*110*	2	6 456	6.01
			IS*200*/IS*605*	2	970	0.9
			IS*3*	1	1 544	1.44
			IS*4*	1	8 46	0.79
			IS*6*	2	2 051	1.91
			IS*66*	1	2 327	2.16
		**Genome (6 134 358 bp)**	**Total**	**101**	**128 336**	**2.09**
BPN29/1	678	chromosome (5 642 477 bp)	BPN29/11115:145	5	6 765	0.12
			IS*21*	2	3 532	0.06
			IS*3*	2	373	0.01
			IS*4*	13	17 738	0.31
			IS*6*	16	14 608	0.26
			IS*607*	6	8 382	0.15
		**Genome (5 717 929 bp)**	**Total**	**44**	**51 398**	**0.9**
BPN36/2	650	chromosome (5 347 123 bp)	IS*200*/IS*605*	2	1 71	0.03
			IS*21*	2	2 662	0.05
			IS*3*	3	1 974	0.04
			IS*4*	10	14 216	0.27
			IS*6*	3	2 515	0.05
			IS*607*	5	8 548	0.16
		plasmid (538 903 bp)	IS*200*/IS*605*	7	9 511	1.76
			IS*256*	1	581	0.11
			IS*3*	2	1 699	0.32
			IS*4*	9	10 921	2.03
			IS*6*	8	8 229	1.53
			IS*630*	1	829,000	0.15
		**Genome (5 886 026 bp)**	**Total**	**53**	**63 395**	**1.07**
BPN36/3	742	chromosome (5 215 878 bp)	IS*200*/IS*605*	4	5 78	0.11
			IS*21*	3	5 326	0.1
			IS*3*	3	1 784	0.03
			IS*4*	8	10 646	0.2
			IS*6*	1	1 184	0.02
			IS*607*	6	11 299	0.22
		plasmid (481 187 bp)	IS*200*/IS*605*	8	8 836	1.84
			IS*3*	2	1 515	0.31
			IS*4*	13	20 841	4.33
			IS*6*	17	15 658	3.25
		**Genome (5 763 140 bp)**	**Total**	**65**	**82 869**	**1.45**
BPN37/1	655	chromosome (5 322 329 bp)	IS*110*	2	3 162	0.06
			IS*200*/IS*605*	2	1 189	0.02
			IS*21*	3	5 254	0,10
			IS*3*	7	7 115	0.13
			IS*4*	2	3 097	0.06
			IS*607*	4	6 951	0.13
			new	1	4 181	0.08
		plasmid (374 906 bp)	IS*200*/IS*605*	7	6 915	1.84
			IS*3*	1	1 345	0.36
			IS*4*	6	8 619	2.3
			IS*6*	9	8 433	2.25
		plasmid (225 867 bp)	IS*3*	1	508	0.22
			IS*4*	4	7 32	3.24
			IS*6*	2	1 607	0.71
		plasmid (54 449 bp)	IS*21*	1	2 347	4.31
		**Genome (6 029 494 bp)**	**Total**	**52**	**68 043**	**1.13**
		chromosome (5 490 503 bp)	IS*200*/IS*605*	5	4 833	0.09
			IS*21*	3	5 433	0.1
			IS*3*	6	5 174	0.09
			IS*4*	9	12 482	0.23
			IS*6*	3	2 64	0.05
			IS*607*	4	7 446	0.14

BPN37/2	662		ISL*3*	1	1 187	0.02
		plasmid (371 005 bp)	IS*200*/IS*605*	5	7 514	2.03
			IS*3*	2	1 032	0.28
			IS*4*	5	5 205	1.4
			IS*6*	5	5 113	1.38
		plasmid (77 113 bp)	IS*3*	2	1 081	1,40
		**Genome (5 956 071 bp)**	**Total**	**50**	**59 14**	**0.99**
BPN43/2	678	chromosome (5 205 885 bp)	IS*200*/IS*605*	2	2 233	0.04
			IS*21*	2	2 826	0.05
			IS*3*	2	373	0.01
			IS*4*	7	10 446	0.2
			IS*6*	3	3 587	0.07
			IS*607*	6	8 382	0.16
		plasmid (400 360 bp)	IS*200*/IS*605*	3	4 008	1,00
			IS*4*	5	5 644	1.41
			IS*6*	13	11 021	2.75
		plasmid (50 285 bp)	IS*4*	1	1 61	3.2
			IS*6*	3	2 491	4.95
		**Genome (5 741 417 bp)**	**Total**	**47**	**52 621**	**0.92**
BPN51/1	667	chromosome (5 281 951 bp)	IS*110*	1	1 807	0.03
			IS*200*/IS*605*	5	6 223	0.12
			IS*21*	2	2 823	0.05
			IS*256*	1	1 437	0.03
			IS*3*	4	5 868	0.11
			IS*4*	6	8 713	0.16
			IS*6*	2	1 99	0.04
			IS607	5	7 558	0.14
		plasmid (318 875 bp)	IS*1595*	1	1 557	0.49
			IS*200*/IS*605*	3	4 161	1.3
			IS*256*	1	585	0.18
			IS*3*	1	1 503	0.47
			IS*4*	7	11 606	3.64
			IS*6*	7	5 094	1.6
		plasmid (123 831 bp)	IS*200*/IS*605*	3	2 503	2.02
			IS*4*	2	1 371	1.11
			IS*6*	1	1 117	0.9
		**Genome (5 779 348 bp)**	**Total**	**52**	**65 916**	**1.14**
BPN52/2	657	chromosome (5 266 779 bp)	IS*110*	1	1 663	0.03
			IS*200*/IS*605*	7	8 688	0.16
			IS*21*	2	2 829	0.05
			IS*256*	1	1 437	0.03
			IS*3*	13	16 591	0.32
			IS*30*	1	243	0.01
			IS*4*	5	4 692	0.09
			IS*6*	3	1 634	0.03
			IS*607*	3	4 829	0.09
		plasmid (479 471 bp)	IS*200*/IS*605*	1	668	0.14
			IS*256*	1	585	0.12
			IS*3*	3	4 048	0.84
			IS*4*	8	8 439	1.76
			IS*6*	11	10 342	2.16
		**Genome (5 798 832 bp)**	**Total**	**60**	**66 688**	**1.15**
BPN54/2	668	chromosome (5 208 286 bp)	IS*1595*	1	842	0.02
			IS*200*/IS*605*	3	4 239	0.08
			IS*21*	3	5 18	0.1
			IS*3*	5	4 121	0.08
			IS*4*	7	10 451	0.2
			IS*6*	7	6 613	0.13
			IS*607*	6	9 578	0.18
		plasmid (419 002 bp)	IS*200*/IS*605*	6	8 08	1.93
			IS*4*	11	15 929	3.8
			IS*6*	15	17 15	4.09
			new	1	2 672	0.64
			IS*200*/IS*605*	5	8 12	3.5

		plasmid (232 193 bp)	IS*3*	1	1 448	0.62
			IS*4*	11	17 159	7.39
			IS*6*	9	7 344	3.16
		plasmid (65 006 bp)	IS*200*/IS*605*	4	4 776	7.35
			IS*4*	2	1 817	2.8
		**Genome (5 956 428 bp)**	**Total**	**97**	**125 519**	**2.11**
BPN57/2	739	chromosome (5 067 631 bp)	IS*1182*	2	1 605	0.03
			IS*200*/IS*605*	7	9 037	0.18
			IS*21*	2	2 527	0.05
			IS*3*	12	15 676	0.31
			IS*4*	5	5 706	0.11
			IS*6*	1	169	0.01
			IS*607*	6	9 664	0.19
		plasmid (369 824 bp)	IS*200*/IS*605*	7	10 722	2.9
			IS*3*	2	1 763	0.48
			IS*4*	4	2 776	0.75
			IS*6*	12	13 461	3.64
			IS*NCY*	1	1 126	0.3
		plasmid (92 054 bp)	IS*200*/IS*605*	4	4 251	4.62
			IS*3*	1	1 316	1.43
			IS*4*	3	6 881	7.47
		**Genome (5 554 158 bp)**	**Total**	**69**	**86 68**	**1.56**
BPN58/4	708	chromosome (5 248 764 bp)	IS*110*	2	4 39	0.08
			IS*200*/IS*605*	3	3 35	0.06
			IS*21*	2	2 649	0.05
			IS*3*	7	10 778	0.21
			IS*4*	5	8 651	0.16
			IS*6*	1	1 284	0.02
			IS*607*	3	5 809	0.11
		plasmid (437 823 bp)	IS*200*/IS*605*	2	2 776	0.63
			IS*21*	1	155	0.04
			IS*3*	4	4 739	1.08
			IS*4*	9	9 605	2.19
			IS*6*	11	8 271	1.89
			IS*L3*	1	2 345	0.54
		plasmid (211 646 bp)	IS*200*/IS*605*	3	2 666	1.26
			IS*6*	1	1 39	0.66
		plasmid (53 401 bp)	IS*21*	1	1 986	3.72
		**Genome (5 951 634 bp)**	**Total**	**56**	**70 844**	**1.19**
BPN102	1507	chromosome (5 093 759 bp)	IS*200*/IS*605*	4	6 129	0.12
			IS*21*	2	2 824	0.06
			IS*3*	9	10 724	0.21
			IS*4*	9	13 78	0.27
			IS*5*	2	3 376	0.07
			IS*6*	7	6 645	0.13
			IS*607*	6	9 579	0.19
		plasmid (357 042 bp)	IS*200*/IS*605*	6	9 361	2.62
			IS*3*	2	2 684	0.75
			IS*4*	3	5 354	1.5
			IS*6*	11	13 743	3.85
		plasmid (231 973 bp)	IS*200*/IS*605*	6	8 417	3.63
			IS*3*	1	1 448	0.62
			IS*4*	9	13 501	5.82
			IS*6*	10	8 776	3.78
		plasmid (54 371 bp)	IS*21*	1	2 083	3.83
		**Genome (5 747 492 bp)**	**Total**	**88**	**118 424**	**2.06**
BPN121	1519	chromosome (5 243 491 bp)	IS*200*/IS*605*	3	4 257	0.08
			IS*21*	2	2 826	0.05
			IS*3*	2	1 607	0.03
			IS*4*	7	7 563	0.14
			IS*6*	1	1 184	0.02
			IS*607*	5	7 86	0.15
		plasmid (396 505 bp)	IS*200*/IS*605*	10	12 057	3.04
			IS*3*	2	1 515	0.38

		plasmid ([illegible] bp)	IS*4*	7	9 795	2.47
			IS*6*	12	10 982	2.77
		plasmid (33 318 bp)	IS*6*	4	3 448	10.35
		**Genome (5 751 434 bp)**	**Total**	**55**	**63 094**	**1.1**
BPN211	742	chromosome (5 265 340 bp)	IS*200*/IS*605*	4	5 78	0.11
			IS*21*	3	4 62	0.09
			IS*3*	3	1 835	0.03
			IS*4*	7	7 445	0.14
			IS*6*	1	1 184	0.02
			IS*607*	5	8 355	0.16
		plasmid (511 214 bp)	IS*200*/IS*605*	8	8 707	1.7
			IS*3*	2	1 515	0.3
			IS*4*	10	12 4	2.43
			IS*6*	14	12 126	2.37
		plasmid (33 318 bp)	IS*6*	4	3 447	10.35
		**Genome (5 809 871 bp)**	**Total**	**61**	**67 414**	**1.16**
BPN401	1568	chromosome (5 369 163 bp)	IS*200*/IS*605*	4	4 491	0.08
			IS*21*	2	2 826	0.05
			IS*256*	1	1 437	0.03
			IS*3*	4	4 945	0.09
			IS*4*	4	12 792	0.24
			IS*6*	3	3 202	0.06
			IS*607*	6	9 317	0.17
		plasmid (394 614 bp)	IS*200*/IS*605*	7	7 888	2,00
			IS*3*	3	2 938	0.74
			IS*4*	6	6 843	1.73
			IS*6*	10	9 872	2.5
		plasmid (37 069 bp)	IS*6*	4	3 495	9.43
		**Genome (5 939 450 bp)**	**Total**	**54**	**70 046**	**1.18**
BPN573	742	chromosome (5 281 925 bp)	IS*200*/IS*605*	4	5 78	0.11
			IS*21*	3	4 62	0.09
			IS*3*	3	1 835	0.03
			IS*4*	8	9 096	0.17
			IS*6*	1	1 184	0.02
			IS*607*	6	10 303	0.2
		plasmid (481 041 bp)	IS*200*/IS*605*	9	8 658	1.8
			IS*3*	2	1 515	0.31
			IS*4*	13	20 846	4.33
			IS*6*	17	15 658	3.26
		**Genome (5 762 966 bp)**	**Total**	**66**	**79 495**	**1.38**
BPN601	1519	chromosome (5 245 175 bp)	IS*200*/IS*605*	3	4 257	0.08
			IS*21*	2	2 826	0.05
			IS*3*	2	1 607	0.03
			IS*4*	7	7 563	0.14
			IS*6*	1	1 184	0.02
			IS*607*	5	7 86	0.15
		plasmid (396 394 bp)	IS*200*/IS*605*	10	12 057	3.04
			IS*3*	2	1 515	0.38
			IS*4*	7	9 795	2.47
			IS*6*	12	10 982	2.77
		plasmid (33 322 bp)	IS*6*	4	3 448	10.35
		**Genome (5 753 119 bp)**	**Total**	**55**	**63 094**	**1.1**
JAS06/1	222	chromosome (5 361 170 bp)	IS*110*	1	1 679	0.03
			IS*200*/IS*605*	2	603	0.01
			IS*21*	3	5 737	0.11
			IS*4*	7	8 779	0.16
			IS*6*	4	2 719	0.05
			IS*607*	2	3 315	0.06
		plasmid (487 389 bp)	IS*200*/IS*605*	6	5 454	1.12
			IS*256*	1	585	0.12
			IS*4*	7	9 697	1.99
			IS*6*	9	10 999	2.26
		plasmid (87 819 bp)	IS*200*/IS*605*	3	1 69	1.92
			IS*5*	2	2 916	3.32

		**Genome (5 938 126 bp)**	**Total**	**47**	**54 173**	**0.91**
JAS06/3	617	chromosome (5 348 830 bp)	IS110	1	1 758	0.03
			IS*200*/IS*605*	2	2 271	0.04
			IS*21*	5	7 738	0.14
			IS*3*	1	213	0.01
			IS*4*	7	10 948	0.2
			IS*6*	4	3 62	0.07
			IS*607*	3	5 669	0.11
		plasmid (233 674 bp)	IS*21*	3	3 403	1.46
			IS*3*	3	3 875	1.66
			IS*4*	12	16 846	7.21
			IS*6*	12	10 664	4.56
			IS*NCY*	1	1 109	0.47
			new	2	1 863	0.8
		plasmid (207 206 bp)	IS*200*/IS*605*	1	1 374	0.66
			IS*21*	2	5 218	2.52
			IS*3*	1	1 345	0.65
			IS*4*	4	7 153	3.45
			IS*6*	2	1 095	0.53
		**Genome (5 865 739 bp)**	**Total**	**66**	**86 162**	**1.47**
JAS12/5	618	chromosome (5 233 313 bp)	IS*1182*	2	3 555	0.07
			IS*200*/IS*605*	4	5 167	0.1
			IS*21*	2	3 532	0.07
			IS*3*	2	2 933	0.06
			IS*4*	5	7 219	0.14
			IS*6*	7	4 2	0.08
			IS*607*	9	14 941	0.29
		plasmid (205 891 bp)	IS*200*/IS*605*	3	3 2	1.55
			IS*4*	7	9 79	4.75
			IS*6*	13	10 44	5.07
		**Genome (5 491 933 bp)**	**Total**	**54**	**64 977**	**1.18**
JAS15/1	624	chromosome (5 291 025 bp)	IS*200*/IS*605*	3	1 908	0.04
			IS*21*	2	2 826	0.05
			IS*3*	5	6 366	0.12
			IS*4*	6	11 276	0.21
			IS*6*	1	204	0.01
			IS*607*	4	7 602	0.14
		plasmid (390 912 bp)	IS*200*/IS*605*	5	6 999	1.79
			IS*3*	2	2 836	0.73
			IS*4*	7	6 735	1.72
			IS*6*	20	17 01	4.35
			new	1	2 711	0.69
		plasmid (208 405 bp)	IS*3*	1	979	0.47
			IS*4*	4	6 342	3.04
			IS*6*	7	6 7	3.21
		plasmid (85 331 bp)	IS*200*/IS*605*	3	2 493	2.92
			IS*4*	2	1 645	1.93
		**Genome (6 010 975 bp)**	**Total**	**73**	**84 632**	**1.41**
JAS23/1	727	chromosome (5 313 017 bp)	IS*110*	1	1 679	0.03
			IS*200*/IS*605*	1	1 305	0.02
			IS*21*	2	2 826	0.05
			IS*3*	2	462	0.01
			IS*4*	7	8 599	0.16
			IS*6*	5	2 324	0.04
			IS*607*	3	5 282	0.1
		plasmid (508 817 bp)	IS*200*/IS*605*	6	5 469	1.07
			IS*256*	1	585	0.11
			IS*4*	10	13 031	2.56
			IS*6*	11	13 391	2.63
		**Genome (5 821 834 bp)**	**Total**	**49**	**54 953**	**0.94**
		chromosome (5 390 204 bp)	IS*110*	2	3 401	0.06
			IS*21*	3	5 19	0.1
			IS*3*	1	249	0.01
			IS*4*	8	9 163	0.17

JAS83/3	615		IS*6*	5	3 713	0.07
			IS*607*	5	8 403	0.16
		plasmid (545 956 bp)	IS*110*	2	3 06	0.56
			IS*1182*	1	1 431	0.26
			IS*200*/IS*605*	49	33 783	6.19
			IS*21*	2	3 162	0.58
			IS*3*	16	18 31	3.35
			IS*4*	7	10 528	1.93
			IS*6*	17	13 736	2.52
			IS*607*	1	187	0.03
		**Genome (6 005 285 bp)**	**Total**	**119**	**114 316**	**1.9**
JAS85/1	410	chromosome (5 202 602 bp)	IS110	1	1 624	0.03
			IS*200*/IS*605*	3	1 683	0.03
			IS*21*	2	3 532	0.07
			IS*3*	2	462	0.01
			IS*4*	6	7 683	0.15
			IS*6*	5	2 509	0.05
			IS*607*	3	5 282	0.1
		plasmid (482 275 bp)	IS*200*/IS*605*	7	7 583	1.57
			IS*256*	1	585	0.12
			IS*4*	6	8 081	1.68
			IS*6*	9	9 415	1.95
		plasmid (136 324 bp)	IS*200*/IS*605*	1	1 111	0.81
		**Genome (5 821 266 bp)**	**Total**	**46**	**49 55**	**0.85**
JAS94/5	726	chromosome (5 275 222 bp)	IS*200*/IS*605*	2	603	0.01
			IS*21*	2	2 826	0.05
			IS*3*	1	249	0.01
			IS*4*	8	10 461	0.2
			IS*6*	3	2 515	0.05
			IS*607*	3	5 584	0.11
		plasmid (347 238 bp)	IS*200*/IS*605*	2	4 968	1.43
			IS256	1	585	0.17
			IS*4*	9	16 21	4.67
			IS*6*	6	5 052	1.45
		**Genome (5 642 620 bp)**	**Total**	**37**	**49 053**	**0.87**
JAS014	1511	chromosome (5 146 518 bp)	IS*110*	1	2 699	0.05
			IS*1595*	1	1 636	0.03
			IS*200*/IS*605*	3	3 997	0.08
			IS*21*	2	2 826	0.05
			IS*3*	3	2 52	0.05
			IS*4*	12	19 337	0.38
			IS*6*	6	4 203	0.08
			IS*607*	5	8 167	0.16
		plasmid (185 643 bp)	IS*200*/IS*605*	4	4 25	2.29
			IS*3*	1	240000	0.13
			IS*4*	5	4 992	2.69
			IS*6*	9	9 499	5.12
		**Genome (5 342 665 bp)**	**Total**	**52**	**64 366**	**1.2**
JAS391*	671	chromosome (5 235 951 bp)	IS*110*	2	3 176	0.06
			IS*200*/IS*605*	3	2 894	0.06
			IS*21*	2	2 649	0.05
			IS*3*	8	10 287	0.2
			IS*4*	5	6 996	0.13
			IS*6*	2	1 074	0.02
			IS*607*	6	9 547	0.18
			new	1	3 145	0.06
		plasmid (405 965 bp)	IS*200*/IS*605*	8	10 294	2.54
			IS*3*	2	2 882	0.71
			IS*4*	3	2 799	0.69
			IS*6*	17	15 408	3.8
			new	1	3 145	0.77
		plasmid (247 123 bp)	IS*110*	1	1 729	0.7
			IS*200*/IS*605*	3	4 164	1.68
			IS*3*	1	1 291	0.52

			IS*4*	3	3 07	1.24
			IS*6*	16	13 472	5.45
		plasmid (109 920 bp)	IS*200*/IS*605*	4	5 014	4.56
			IS*4*	2	1 376	1.25
			IS*6*	1	1 593	1.45
		**Genome (6 056 335 bp)**	**Total**	**91**	**106 005**	**1.75**
JAS481*	624	chromosome (5 296 574 bp)	IS*200*/IS*605*	1	1 305	0.02
			IS*21*	2	2 826	0.05
			IS*3*	7	9 042	0.17
			IS*4*	8	12 338	0.23
			IS*6*	5	2 902	0.05
			IS*607*	4	7 119	0.13
		plasmid (334 352 bp)	IS*200*/IS*605*	5	5 346	1.6
			IS*4*	4	2 97	0.89
			IS*6*	20	16 563	4.95
		plasmid (237 265 bp)	IS*256*	1	917	0.39
			IS*3*	1	979	0.41
			IS*4*	7	14 079	5.93
			IS*6*	10	11 904	5.02
		plasmid (124 309 bp)	IS*200*/IS*605*	2	976	0.79
			IS*3*	1	1 326	1.07
			IS*4*	3	4 03	3.24
			IS*6*	2	1 968	1.58
			IS*66*	1	2 327	1.87
		**Genome (6 014 015 bp)**	**Total**	**84**	98 917	**1.64**
JAS635	695	chromosome (5 473 370 bp)	JAS6354497:513	1	1 752	0.03
			IS*200*/IS*605*	3	5 139	0.09
			IS*21*	4	7 685	0.14
			IS*3*	1	919	0.02
			IS*4*	4	3 238	0.06
			IS*6*	4	4 571	0.08
			IS*607*	3	5 745	0.1
			IS*L3*	3	5 58	0.1
		plasmid (192 434 bp)	IS*21*	1	2 131	1.11
			IS*4*	6	12 301	6.39
			IS*6*	16	13 018	6.76
			IS*3*	1	1 864	0.97
			IS*NCY*	1	966	0.5
		plasmid (97 153 bp)	IS*NCY*	1	1 139	1.17
		plasmid (73 718 bp)	IS*200*/IS*605*	1	1 307	1.77
			IS*607*	1	2 2	2.98
		plasmid (70 690 bp)	IS*4*	1	1 657	2.34
		plasmid (48 338 bp)	IS*110*	1	1 483	3.07
			IS*4*	1	1 289	2.67
			IS*6*	2	1 778	3.68
			IS*630*	1	329	0.68
		**Genome (6 163 981 bp)**	**Total**	**57**	**76 091**	**1.23**
JAS823	1514	chromosome (5 114 971 bp)	IS*110*	1	2 965	0.06
			IS*1595*	1	1 156	0.02
			IS*200*/IS*605*	4	5 829	0.11
			IS*21*	2	2 639	0.05
			IS*3*	7	8 14	0.16
			IS*4*	7	11 47	0.22
			IS*6*	7	4 886	0.1
			IS*607*	3	4 98	0.1
		plasmid (162 314 bp)	IS*110*	1	2 039	1.26
			IS*1182*	1	1 034	0.64
			IS*200*/IS*605*	4	3 126	1.93
			IS*3*	1	240	0.15
			IS*4*	9	10 879	6.7
			IS*6*	5	6 577	4.05
		plasmid (52 218 bp)	IS*607*	1	1 688	3.23
		**Genome (5 344 184 bp)**	**Total**	**54**	**67 648**	**1.27**
			IS*110*	1	1 752	0.03

JAS1004	695	chromosome (5 407 642 bp)	IS*200*/IS*605*	3	5 139	0.1
			IS*21*	4	6 979	0.13
			IS*3*	1	919	0.02
			IS*4*	4	3 238	0.06
			IS*6*	4	4 571	0.08
			IS*607*	3	5 745	0.11
		plasmid (506 603 bp)	IS*200*/IS*605*	8	9 317	1.84
			IS*3*	2	2 543	0.5
			IS*4*	10	8 255	1.63
			IS*6*	8	8 007	1.58
		plasmid (198 488 bp)	IS*1182*	1	769	0.39
			IS*3*	3	4 274	2.15
			IS*4*	13	17 408	8.77
			IS*6*	17	13 916	7.01
			IS*630*	1	1 167	0.59
			IS*NCY*	1	1 002	0.5
			new	1	2 651	1.34
		plasmid (77 756 bp)	IS*607*	1	2 2	2.83
		plasmid (48 337 bp)	IS*110*	1	1 483	3.07
			IS*4*	1	1 289	2.67
			IS*6*	2	1 778	3.68
			IS*630*	1	329	0.68
		plasmid (13 386 bp)	IS*4*	1	2 077	15.52
		**Genome (6 338 271 bp)**	**Total**	**92**	**106 808**	**1.69**

Legend:

***Bacillus mycoides* isolate**, isolates from soil samples collected in Białowieża National Park are indicated as BPN, while from soil samples picked up in a farmland in Jasienowka are marked as JAS; isolates forming rhizoidal colonies on agar plates are indicated with an asterisk;

ST, sequence type determined with the use of Multilocus Sequence Types (MLST). For details see Drewnowska and Swiecicka [2013], and https://pubmlst.org/organisms/bacillus-cereus;

Replicon, a DNA molecule, either a chromosome or a plasmid with an IS;

IS location, a contig (a chromsome or a plasmid) containing an IS sequence; a length of the contig is given in parantheses;

IS family, a family to which an IS was classified;

No. of ISs, a number of ISs present in a replicon;

Total length [bp], a total length of ISs in a replicon [bp];

Part of a replicon [%], a percentage of either a chromosome or a plasmid constituting ISs;

Table S6 Pairwise average nucleotide identity (ANI) values.

Strain	*B. mycoides* BPN601	*B. mycoides* BPN58/4	*B. mycoides* JAS06/1	*B. wiedmannii* FSL W8-0169	*B. cereus* ATCC 14579	*B. cytotoxicus* NVH 391-98	*B. mycoides* JAS014	*B. mycoides* BPN36/3	*B. thuringiensis* ATCC 10792	*B. mycoides* JAS12/5	*B. mycoides* BPN211	*B. mycoides* BPN573
B. mycoides BPN601	100,00%	97,14%	97,75%	90,59%	89,93%	85,18%	94,89%	99,48%	89,97%	97,28%	99,46%	99,48%
B. mycoides BPN58/4	97,14%	100,00%	97,14%	90,46%	89,94%	85,19%	95,07%	97,12%	89,92%	96,79%	97,13%	97,11%
B. mycoides JAS06/1	97,75%	97,14%	100,00%	90,61%	89,90%	85,18%	95,00%	97,76%	89,93%	97,48%	97,79%	97,76%
B. wiedmannii FSL W8-0169	90,59%	90,46%	90,61%	100,00%	91,62%	85,08%	90,15%	90,57%	91,65%	90,58%	90,58%	90,57%
B. cereus ATCC 14579	89,93%	89,94%	89,90%	91,62%	100,00%	85,23%	89,77%	89,90%	96,81%	89,92%	89,92%	89,91%
B. cytotoxicus NVH 391-98	85,18%	85,19%	85,18%	85,08%	85,23%	100,00%	85,23%	85,22%	85,22%	85,19%	85,22%	85,22%
B. mycoides JAS014	94,89%	95,07%	95,00%	90,15%	89,77%	85,23%	100,00%	94,89%	89,80%	94,89%	94,87%	94,89%
B. mycoides BPN36/3	99,48%	97,12%	97,76%	90,57%	89,90%	85,22%	94,89%	100,00%	89,99%	97,26%	99,78%	99,99%
B. thuringiensis ATCC 10792	89,97%	89,92%	89,93%	91,65%	96,81%	85,22%	89,80%	89,99%	100,00%	89,97%	89,98%	89,97%
B. mycoides JAS12/5	97,28%	96,79%	97,48%	90,58%	89,92%	85,19%	94,89%	97,26%	89,97%	100,00%	97,25%	97,26%
B. mycoides BPN211	99,46%	97,13%	97,79%	90,58%	89,92%	85,22%	94,87%	99,78%	89,98%	97,25%	100,00%	99,78%
B. mycoides BPN573	99,48%	97,11%	97,76%	90,57%	89,91%	85,22%	94,89%	99,99%	89,97%	97,26%	99,78%	100,00%
B. toyonensis BCT-7112	90,87%	90,90%	90,95%	91,61%	91,82%	85,15%	90,66%	90,89%	91,86%	90,83%	90,90%	90,90%
B. mycoides BPN54/2	95,08%	95,26%	95,17%	90,14%	89,78%	85,15%	97,29%	95,11%	89,79%	95,02%	95,09%	95,11%
B. anthracis Ames	89,93%	89,88%	89,92%	93,29%	91,90%	85,25%	89,75%	89,90%	91,66%	89,95%	89,93%	89,91%
B. mycoides JAS94/5	97,77%	97,17%	99,49%	90,64%	89,92%	85,18%	95,03%	97,78%	89,91%	97,49%	97,81%	97,78%
B. mycoides JAS83/3	97,71%	97,10%	99,17%	90,57%	89,90%	85,17%	94,95%	97,72%	89,87%	97,42%	97,74%	97,72%
B. mycoides JAS391	97,83%	97,05%	97,98%	90,66%	89,93%	85,18%	94,96%	97,84%	89,97%	97,52%	97,84%	97,84%
B. mycoides BPN29/1	97,86%	96,98%	97,75%	90,61%	89,90%	85,20%	94,89%	97,85%	89,93%	97,47%	97,83%	97,85%
B. mycoides BPN43/2	97,87%	97,00%	97,76%	90,62%	89,90%	85,22%	94,89%	97,86%	89,96%	97,47%	97,83%	97,86%
B. mycoides BPN37/2	98,02%	97,01%	98,20%	90,66%	89,95%	85,20%	94,82%	98,06%	89,99%	97,36%	98,07%	98,06%
B. mycoides BPN57/2	95,54%	95,62%	95,56%	90,22%	89,69%	85,32%	95,93%	95,52%	89,71%	95,38%	95,51%	95,53%
B. mycoides BPN36/2	97,75%	97,16%	99,36%	90,63%	89,94%	85,16%	95,00%	97,79%	89,93%	97,49%	97,80%	97,79%
B. mycoides BPN401	97,97%	97,07%	97,94%	90,64%	89,88%	85,21%	94,86%	97,94%	89,92%	97,50%	97,96%	97,94%
B. mycoides BPN37/1	97,72%	97,15%	99,09%	90,58%	89,88%	85,13%	94,96%	97,77%	89,89%	97,41%	97,77%	97,77%
B. mycoides JAS15/1	97,74%	97,11%	99,03%	90,61%	89,95%	85,22%	94,98%	97,79%	89,94%	97,37%	97,81%	97,79%
B. mycoides JAS481	97,71%	97,10%	99,07%	90,65%	89,93%	85,23%	95,01%	97,77%	89,92%	97,40%	97,81%	97,77%
B. mycoides JAS635	97,82%	96,89%	97,66%	90,70%	89,93%	85,19%	94,87%	97,84%	89,94%	97,51%	97,85%	97,84%
B. thuringiensis HD1	90,01%	90,01%	89,99%	91,71%	97,12%	85,26%	89,83%	90,02%	96,37%	89,99%	90,03%	90,02%
B. mycoides BPN07/3	95,07%	95,27%	95,18%	90,15%	89,76%	85,16%	97,32%	95,10%	89,76%	95,02%	95,09%	95,10%
B. mycoides BPN08/1	97,71%	97,13%	99,07%	90,60%	89,90%	85,17%	94,95%	97,74%	89,90%	97,37%	97,75%	97,74%
B. mycoides ATCC 6462	97,71%	97,10%	98,99%	90,70%	89,95%	85,22%	94,99%	97,75%	89,93%	97,36%	97,80%	97,75%
B. mycoides JAS823	94,91%	95,08%	95,01%	90,13%	89,77%	85,23%	99,15%	94,93%	89,74%	94,91%	94,92%	94,93%
B. mycoides BPN51/1	97,96%	97,03%	97,85%	90,63%	89,88%	85,24%	94,91%	97,94%	89,90%	97,48%	97,94%	97,94%
B. subtilis str. 168	83,20%	83,53%	83,29%	83,61%	84,00%	83,79%	83,50%	83,26%	84,64%	83,32%	83,21%	83,26%
B. mycoides JAS06/3	97,87%	96,98%	97,78%	90,69%	89,94%	85,19%	94,85%	97,87%	90,01%	97,51%	97,87%	97,87%
B. cereus ATCC 10987	89,93%	89,92%	89,96%	93,48%	91,87%	85,29%	89,76%	89,95%	91,63%	89,96%	89,95%	89,95%
B. mycoides BPN09/1	97,74%	97,14%	99,05%	90,61%	89,92%	85,20%	94,95%	97,77%	89,92%	97,38%	97,80%	97,77%
B. mycoides JAS1004	97,83%	96,89%	97,68%	90,70%	89,93%	85,19%	94,87%	97,85%	89,95%	97,51%	97,85%	97,85%
B. mycoides BPN52/2	97,93%	97,03%	97,74%	90,65%	89,92%	85,15%	94,94%	97,91%	89,91%	97,57%	97,92%	97,91%
B. mycoides JAS85/1	97,71%	97,09%	99,18%	90,63%	89,95%	85,18%	94,98%	97,73%	89,95%	97,47%	97,75%	97,73%
B. mycoides BPN102	95,08%	95,28%	95,15%	90,15%	89,75%	85,18%	97,33%	95,09%	89,79%	95,01%	95,09%	95,09%
B. mycoides BPN03/1	95,04%	95,20%	95,08%	90,10%	89,75%	85,16%	97,22%	95,03%	89,74%	95,02%	95,02%	95,03%
B. mycoides KBAB4	97,83%	96,90%	97,79%	90,64%	89,92%	85,24%	94,90%	97,84%	89,93%	97,48%	97,84%	97,84%
B. mycoides DSM 11821	97,84%	96,98%	97,82%	90,66%	89,83%	85,12%	94,90%	97,84%	89,95%	97,54%	97,84%	97,84%
B. mycoides BPN121	99,99%	97,14%	97,76%	90,58%	89,92%	85,18%	94,88%	99,48%	89,95%	97,28%	99,46%	99,48%
B. mycoides JAS23/1	97,73%	97,09%	99,20%	90,63%	89,92%	85,21%	95,01%	97,77%	89,96%	97,48%	97,79%	97,77%

Strain	*B. toyonensis* BCT-7112	*B. mycoides* BPN54/2	*B. anthracis* Ames	*B. mycoides* JAS94/5	*B. mycoides* JAS83/3	*B. mycoides* JAS31	*B. mycoides* BPN29/1	*B. mycoides* BPN43/2	*B. mycoides* BPN37/2	*B. mycoides* BPN57/2	*B. mycoides* BPN36/2	*B. mycoides* BPN401
B. mycoides BPN601	90,87%	95,08%	89,93%	97,77%	97,71%	97,83%	97,86%	97,87%	98,02%	95,54%	97,75%	97,97%
B. mycoides BPN58/4	90,90%	95,26%	89,88%	97,17%	97,10%	97,05%	96,98%	97,00%	97,01%	95,62%	97,16%	97,07%
B. mycoides JAS06/1	90,95%	95,17%	89,92%	99,49%	99,17%	97,98%	97,75%	97,76%	98,20%	95,56%	99,36%	97,94%
B. wiedmannii FSL W8-0169	91,61%	90,14%	93,29%	90,64%	90,57%	90,66%	90,61%	90,62%	90,66%	90,22%	90,63%	90,64%
B. cereus ATCC 14579	91,82%	89,78%	91,90%	89,92%	89,90%	89,93%	89,90%	89,90%	89,95%	89,69%	89,94%	89,88%
B. cytotoxicus NVH 391-98	85,15%	85,15%	85,25%	85,18%	85,17%	85,18%	85,20%	85,22%	85,20%	85,32%	85,16%	85,21%
B. mycoides JAS014	90,66%	97,29%	89,75%	95,03%	94,95%	94,96%	94,89%	94,89%	94,82%	95,93%	95,00%	94,86%
B. mycoides BPN36/3	90,89%	95,11%	89,90%	97,78%	97,72%	97,84%	97,85%	97,86%	98,06%	95,52%	97,79%	97,94%
B. thuringiensis ATCC 10792	91,86%	89,79%	91,66%	89,91%	89,87%	89,97%	89,93%	89,96%	89,99%	89,71%	89,93%	89,92%
B. mycoides JAS12/5	90,83%	95,02%	89,95%	97,49%	97,42%	97,52%	97,47%	97,47%	97,36%	95,38%	97,49%	97,50%
B. mycoides BPN211	90,90%	95,09%	89,93%	97,81%	97,74%	97,84%	97,83%	97,83%	98,07%	95,51%	97,80%	97,96%
B. mycoides BPN573	90,90%	95,11%	89,91%	97,78%	97,72%	97,84%	97,85%	97,86%	98,06%	95,53%	97,79%	97,94%
B. toyonensis BCT-7112	100,00%	90,61%	91,32%	90,95%	90,93%	90,84%	90,83%	90,82%	90,93%	90,57%	90,96%	90,81%
B. mycoides BPN54/2	90,61%	100,00%	89,75%	95,13%	95,06%	95,10%	95,04%	95,07%	94,96%	96,21%	95,15%	95,10%
B. anthracis Ames	91,32%	89,75%	100,00%	89,91%	89,86%	89,98%	89,92%	89,92%	89,98%	89,67%	89,91%	89,92%
B. mycoides JAS94/5	90,95%	95,13%	89,91%	100,00%	99,26%	98,01%	97,79%	97,80%	98,25%	95,57%	99,43%	97,96%
B. mycoides JAS83/3	90,93%	95,06%	89,86%	99,26%	100,00%	97,94%	97,70%	97,72%	98,20%	95,51%	99,25%	97,90%
B. mycoides JAS391	90,84%	95,10%	89,98%	98,01%	97,94%	100,00%	98,01%	98,02%	97,91%	95,53%	98,04%	98,13%
B. mycoides BPN29/1	90,83%	95,04%	89,92%	97,79%	97,70%	98,01%	100,00%	99,95%	97,90%	95,47%	97,76%	98,58%
B. mycoides BPN43/2	90,82%	95,07%	89,92%	97,80%	97,72%	98,02%	99,95%	100,00%	97,94%	95,48%	97,77%	98,59%
B. mycoides BPN37/2	90,93%	94,96%	89,98%	98,25%	98,20%	97,91%	97,90%	97,94%	100,00%	95,46%	98,23%	98,05%
B. mycoides BPN57/2	90,57%	96,21%	89,67%	95,57%	95,51%	95,53%	95,47%	95,48%	95,46%	100,00%	95,57%	95,54%
B. mycoides BPN36/2	90,96%	95,15%	89,91%	99,43%	99,25%	98,04%	97,76%	97,77%	98,23%	95,57%	100,00%	97,96%
B. mycoides BPN401	90,81%	95,10%	89,92%	97,96%	97,90%	98,13%	98,58%	98,59%	98,05%	95,54%	97,96%	100,00%
B. mycoides BPN37/1	90,93%	95,07%	89,89%	99,15%	99,08%	97,98%	97,72%	97,75%	98,15%	95,56%	99,13%	97,91%
B. mycoides JAS15/1	90,92%	95,14%	89,96%	99,02%	99,07%	98,07%	97,72%	97,73%	98,16%	95,54%	99,07%	97,88%
B. mycoides JAS481	90,93%	95,15%	89,92%	99,11%	99,17%	98,04%	97,68%	97,68%	98,19%	95,52%	99,15%	97,84%
B. mycoides JAS635	90,88%	95,03%	89,92%	97,73%	97,60%	98,11%	98,37%	98,40%	97,83%	95,44%	97,71%	98,34%
B. thuringiensis HD1	91,84%	89,82%	91,82%	90,01%	89,95%	90,01%	89,96%	89,98%	90,05%	89,78%	90,01%	89,95%
B. mycoides BPN07/3	90,60%	98,91%	89,72%	95,15%	95,08%	95,13%	95,05%	95,07%	95,00%	96,19%	95,18%	95,08%
B. mycoides BPN08/1	90,90%	95,11%	89,90%	99,07%	99,12%	97,95%	97,71%	97,72%	98,16%	95,56%	99,13%	97,86%
B. mycoides ATCC 6462	90,91%	95,16%	89,93%	99,05%	99,08%	98,02%	97,67%	97,70%	98,12%	95,52%	99,06%	97,86%
B. mycoides JAS823	90,62%	97,27%	89,71%	95,04%	94,97%	94,96%	94,90%	94,90%	94,85%	95,93%	95,02%	94,90%
B. mycoides BPN51/1	90,82%	95,13%	89,90%	97,89%	97,79%	98,16%	98,58%	98,58%	98,02%	95,50%	97,87%	99,22%
B. subtilis str. 168	83,69%	83,30%	83,49%	83,10%	83,26%	83,03%	82,89%	82,89%	83,37%	84,21%	83,07%	83,13%
B. mycoides JAS06/3	90,89%	95,02%	89,95%	97,81%	97,70%	98,22%	98,23%	98,24%	97,92%	95,52%	97,80%	98,22%
B. cereus ATCC 10987	91,25%	89,74%	94,57%	90,00%	89,93%	90,02%	89,99%	89,98%	90,01%	89,67%	89,98%	89,95%
B. mycoides BPN09/1	90,90%	95,11%	89,92%	99,07%	99,11%	98,03%	97,70%	97,71%	98,22%	95,51%	99,12%	97,86%
B. mycoides JAS1004	90,88%	95,05%	89,92%	97,73%	97,61%	98,13%	98,39%	98,40%	97,86%	95,44%	97,72%	98,34%
B. mycoides BPN52/2	90,88%	95,07%	89,91%	97,77%	97,69%	98,15%	98,33%	98,37%	97,87%	95,57%	97,77%	98,44%
B. mycoides JAS85/1	90,94%	95,15%	89,93%	99,24%	99,17%	98,01%	97,74%	97,75%	98,22%	95,53%	99,20%	97,92%
B. mycoides BPN102	90,59%	99,52%	89,73%	95,15%	95,07%	95,11%	95,05%	95,06%	95,00%	96,19%	95,15%	95,08%
B. mycoides BPN03/1	90,57%	98,12%	89,66%	95,08%	95,03%	95,12%	95,00%	95,01%	94,97%	96,24%	95,08%	95,03%
B. mycoides KBAB4	90,85%	95,05%	90,01%	97,80%	97,74%	98,22%	98,12%	98,17%	97,95%	95,56%	97,80%	98,19%
B. mycoides DSM 11821	90,78%	95,07%	89,86%	97,83%	97,74%	98,27%	98,08%	98,10%	97,87%	95,48%	97,84%	98,19%
B. mycoides BPN121	90,86%	95,08%	89,92%	97,77%	97,72%	97,84%	97,86%	97,88%	98,03%	95,55%	97,75%	97,97%
B. mycoides JAS23/1	90,96%	95,17%	89,92%	99,25%	99,15%	98,03%	97,74%	97,75%	98,25%	95,56%	99,22%	97,91%

Strain	*B. mycoides* BPN37/1	*B. mycoides* JAS15/1	*B. mycoides* JAS481	*B. mycoides* JAS635	*B. thuringiensis* HD1	*B. mycoides* BPN07/3	*B. mycoides* BPN08/1	*B. mycoides* ATCC 6462	*B. mycoides* JAS823	*B. mycoides* BPN51/1	*B. subtilis* str. 168	*B. mycoides* JAS06/3
B. mycoides BPN601	97,72%	97,74%	97,71%	97,82%	90,01%	95,07%	97,71%	97,71%	94,91%	97,96%	83,20%	97,87%
B. mycoides BPN58/4	97,15%	97,11%	97,10%	96,89%	90,01%	95,27%	97,13%	97,10%	95,08%	97,03%	83,53%	96,98%
B. mycoides JAS06/1	99,09%	99,03%	99,07%	97,66%	89,99%	95,18%	99,07%	98,99%	95,01%	97,85%	83,29%	97,78%
B. wiedmannii FSL W8-0169	90,58%	90,61%	90,65%	90,70%	91,71%	90,15%	90,60%	90,70%	90,13%	90,63%	83,61%	90,69%
B. cereus ATCC 14579	89,88%	89,95%	89,93%	89,93%	97,12%	89,76%	89,90%	89,95%	89,77%	89,88%	84,00%	89,94%
B. cytotoxicus NVH 391-98	85,13%	85,22%	85,23%	85,19%	85,26%	85,16%	85,17%	85,22%	85,23%	85,24%	83,79%	85,19%
B. mycoides JAS014	94,96%	94,98%	95,01%	94,87%	89,83%	97,32%	94,95%	94,99%	99,15%	94,91%	83,50%	94,85%
B. mycoides BPN36/3	97,77%	97,79%	97,77%	97,84%	90,02%	95,10%	97,74%	97,75%	94,93%	97,94%	83,26%	97,87%
B. thuringiensis ATCC 10792	89,89%	89,94%	89,92%	89,94%	96,37%	89,76%	89,90%	89,93%	89,74%	89,90%	84,64%	90,01%
B. mycoides JAS12/5	97,41%	97,37%	97,40%	97,51%	89,99%	95,02%	97,37%	97,36%	94,91%	97,48%	83,32%	97,51%
B. mycoides BPN211	97,77%	97,81%	97,81%	97,85%	90,03%	95,09%	97,75%	97,80%	94,92%	97,94%	83,21%	97,87%
B. mycoides BPN573	97,77%	97,79%	97,77%	97,84%	90,02%	95,10%	97,74%	97,75%	94,93%	97,94%	83,26%	97,87%
B. toyonensis BCT-7112	90,93%	90,92%	90,93%	90,88%	91,84%	90,60%	90,90%	90,91%	90,62%	90,82%	83,69%	90,89%
B. mycoides BPN54/2	95,07%	95,14%	95,15%	95,03%	89,82%	98,91%	95,11%	95,16%	97,27%	95,13%	83,30%	95,02%
B. anthracis Ames	89,89%	89,96%	89,92%	89,92%	91,82%	89,72%	89,90%	89,93%	89,71%	89,90%	83,49%	89,95%
B. mycoides JAS94/5	99,15%	99,02%	99,11%	97,73%	90,01%	95,15%	99,07%	99,05%	95,04%	97,89%	83,10%	97,81%
B. mycoides JAS83/3	99,08%	99,07%	99,17%	97,60%	89,95%	95,08%	99,12%	99,08%	94,97%	97,79%	83,26%	97,70%
B. mycoides JAS391	97,98%	98,07%	98,04%	98,11%	90,01%	95,13%	97,95%	98,02%	94,96%	98,16%	83,03%	98,22%
B. mycoides BPN29/1	97,72%	97,72%	97,68%	98,37%	89,96%	95,05%	97,71%	97,67%	94,90%	98,58%	82,89%	98,23%
B. mycoides BPN43/2	97,75%	97,73%	97,68%	98,40%	89,98%	95,07%	97,72%	97,70%	94,90%	98,58%	82,89%	98,24%
B. mycoides BPN37/2	98,15%	98,16%	98,19%	97,83%	90,05%	95,00%	98,16%	98,12%	94,85%	98,02%	83,37%	97,92%
B. mycoides BPN57/2	95,56%	95,54%	95,52%	95,44%	89,78%	96,19%	95,56%	95,52%	95,93%	95,50%	84,21%	95,52%
B. mycoides BPN36/2	99,13%	99,07%	99,15%	97,71%	90,01%	95,18%	99,13%	99,06%	95,02%	97,87%	83,07%	97,80%
B. mycoides BPN401	97,91%	97,88%	97,84%	98,34%	89,95%	95,08%	97,86%	97,86%	94,90%	99,22%	83,13%	98,22%
B. mycoides BPN37/1	100,00%	99,03%	99,11%	97,62%	89,96%	95,11%	99,24%	99,07%	94,98%	97,85%	83,58%	97,74%
B. mycoides JAS15/1	99,03%	100,00%	99,40%	97,74%	90,02%	95,14%	99,04%	99,41%	95,00%	97,88%	82,97%	97,75%
B. mycoides JAS481	99,11%	99,40%	100,00%	97,71%	90,05%	95,17%	99,05%	99,51%	95,01%	97,83%	83,06%	97,75%
B. mycoides JAS635	97,62%	97,74%	97,71%	100,00%	89,98%	95,04%	97,61%	97,71%	94,88%	98,48%	83,09%	98,37%
B. thuringiensis HD1	89,96%	90,02%	90,05%	89,98%	100,00%	89,79%	89,98%	90,01%	89,80%	89,94%	84,43%	90,03%
B. mycoides BPN07/3	95,11%	95,14%	95,17%	95,04%	89,79%	100,00%	95,13%	95,20%	97,32%	95,12%	83,35%	95,02%
B. mycoides BPN08/1	99,24%	99,04%	99,05%	97,61%	89,98%	95,13%	100,00%	99,02%	94,96%	97,80%	83,50%	97,73%
B. mycoides ATCC 6462	99,07%	99,41%	99,51%	97,71%	90,01%	95,20%	99,02%	100,00%	94,99%	97,87%	83,47%	97,73%
B. mycoides JAS823	94,98%	95,00%	95,01%	94,88%	89,80%	97,32%	94,96%	94,99%	100,00%	94,93%	83,68%	94,89%
B. mycoides BPN51/1	97,85%	97,88%	97,83%	98,48%	89,94%	95,12%	97,80%	97,87%	94,93%	100,00%	82,94%	98,25%
B. subtilis str. 168	83,58%	82,97%	83,06%	83,09%	84,43%	83,35%	83,50%	83,47%	83,68%	82,94%	100,00%	83,32%
B. mycoides JAS06/3	97,74%	97,75%	97,75%	98,37%	90,03%	95,02%	97,73%	97,73%	94,89%	98,25%	83,32%	100,00%
B. cereus ATCC 10987	89,95%	89,98%	89,95%	89,99%	91,84%	89,74%	89,97%	89,96%	89,74%	89,93%	84,41%	90,02%
B. mycoides BPN09/1	99,14%	99,41%	99,42%	97,73%	89,99%	95,10%	99,14%	99,29%	94,95%	97,87%	83,00%	97,73%
B. mycoides JAS1004	97,63%	97,75%	97,71%	99,96%	89,98%	95,06%	97,62%	97,72%	94,88%	98,48%	83,17%	98,37%
B. mycoides BPN52/2	97,70%	97,74%	97,72%	98,47%	90,02%	95,08%	97,70%	97,70%	94,95%	98,44%	83,13%	98,48%
B. mycoides JAS85/1	99,08%	99,01%	99,10%	97,72%	90,03%	95,15%	99,01%	99,04%	95,00%	97,83%	82,99%	97,75%
B. mycoides BPN102	95,10%	95,15%	95,18%	95,06%	89,81%	98,99%	95,10%	95,19%	97,28%	95,12%	83,45%	95,01%
B. mycoides BPN03/1	95,08%	95,12%	95,13%	94,96%	89,77%	98,10%	95,07%	95,15%	97,22%	95,05%	83,06%	94,99%
B. mycoides KBAB4	97,74%	97,79%	97,74%	98,22%	89,98%	95,06%	97,73%	97,72%	94,94%	98,19%	83,02%	98,44%
B. mycoides DSM 11821	97,78%	97,73%	97,76%	98,28%	89,91%	95,05%	97,72%	97,70%	94,93%	98,23%	83,52%	98,62%
B. mycoides BPN121	97,72%	97,75%	97,72%	97,83%	89,99%	95,08%	97,69%	97,72%	94,91%	97,97%	83,25%	97,87%
B. mycoides JAS23/1	99,09%	99,04%	99,12%	97,74%	90,02%	95,18%	99,03%	99,07%	95,03%	97,84%	83,01%	97,80%

Strain	*B. cereus* ATCC 10987	*B. mycoides* BPN09/1	*B. mycoides* JAS1004	*B. mycoides* BPN52/2	*B. mycoides* JAS85/1	*B. mycoides* BPN102	*B. mycoides* BPN03/1	*B. mycoides* KBAB4	*B. mycoides* DSM 11821	*B. mycoides* BPN121	*B. mycoides* JAS23/1
B. mycoides BPN601	89,93%	97,74%	97,83%	97,93%	97,71%	95,08%	95,04%	97,83%	97,84%	99,99%	97,73%
B. mycoides BPN58/4	89,92%	97,14%	96,89%	97,03%	97,09%	95,28%	95,20%	96,90%	96,98%	97,14%	97,09%
B. mycoides JAS06/1	89,96%	99,05%	97,68%	97,74%	99,18%	95,15%	95,08%	97,79%	97,82%	97,76%	99,20%
B. wiedmannii FSL W8-0169	93,48%	90,61%	90,70%	90,65%	90,63%	90,15%	90,10%	90,64%	90,66%	90,58%	90,63%
B. cereus ATCC 14579	91,87%	89,92%	89,93%	89,92%	89,95%	89,75%	89,75%	89,92%	89,83%	89,92%	89,92%
B. cytotoxicus NVH 391-98	85,29%	85,20%	85,19%	85,15%	85,18%	85,18%	85,16%	85,24%	85,12%	85,18%	85,21%
B. mycoides JAS014	89,76%	94,95%	94,87%	94,94%	94,98%	97,33%	97,22%	94,90%	94,90%	94,88%	95,01%
B. mycoides BPN36/3	89,95%	97,77%	97,85%	97,91%	97,73%	95,09%	95,03%	97,84%	97,84%	99,48%	97,77%
B. thuringiensis ATCC 10792	91,63%	89,92%	89,95%	89,91%	89,95%	89,79%	89,74%	89,93%	89,95%	89,95%	89,96%
B. mycoides JAS12/5	89,96%	97,38%	97,51%	97,57%	97,47%	95,01%	95,02%	97,48%	97,54%	97,28%	97,48%
B. mycoides BPN211	89,95%	97,80%	97,85%	97,92%	97,75%	95,09%	95,02%	97,84%	97,84%	99,46%	97,79%
B. mycoides BPN573	89,95%	97,77%	97,85%	97,91%	97,73%	95,09%	95,03%	97,84%	97,84%	99,48%	97,77%
B. toyonensis BCT-7112	91,25%	90,90%	90,88%	90,88%	90,94%	90,59%	90,57%	90,85%	90,78%	90,86%	90,96%
B. mycoides BPN54/2	89,74%	95,11%	95,05%	95,07%	95,15%	99,52%	98,12%	95,05%	95,07%	95,08%	95,17%
B. anthracis Ames	94,57%	89,92%	89,92%	89,91%	89,93%	89,73%	89,66%	90,01%	89,86%	89,92%	89,92%
B. mycoides JAS94/5	90,00%	99,07%	97,73%	97,77%	99,24%	95,15%	95,08%	97,80%	97,83%	97,77%	99,25%
B. mycoides JAS83/3	89,93%	99,11%	97,61%	97,69%	99,17%	95,07%	95,03%	97,74%	97,74%	97,72%	99,15%
B. mycoides JAS391	90,02%	98,03%	98,13%	98,15%	98,01%	95,11%	95,12%	98,22%	98,27%	97,84%	98,03%
B. mycoides BPN29/1	89,99%	97,70%	98,39%	98,33%	97,74%	95,05%	95,00%	98,12%	98,08%	97,86%	97,74%
B. mycoides BPN43/2	89,98%	97,71%	98,40%	98,37%	97,75%	95,06%	95,01%	98,17%	98,10%	97,88%	97,75%
B. mycoides BPN37/2	90,01%	98,22%	97,86%	97,87%	98,22%	95,00%	94,97%	97,95%	97,87%	98,03%	98,25%
B. mycoides BPN57/2	89,67%	95,51%	95,44%	95,57%	95,53%	96,19%	96,24%	95,56%	95,48%	95,55%	95,56%
B. mycoides BPN36/2	89,98%	99,12%	97,72%	97,77%	99,20%	95,15%	95,08%	97,80%	97,84%	97,75%	99,22%
B. mycoides BPN401	89,95%	97,86%	98,34%	98,44%	97,92%	95,08%	95,03%	98,19%	98,19%	97,97%	97,91%
B. mycoides BPN37/1	89,95%	99,14%	97,63%	97,70%	99,08%	95,10%	95,08%	97,74%	97,78%	97,72%	99,09%
B. mycoides JAS15/1	89,98%	99,41%	97,75%	97,74%	99,01%	95,15%	95,12%	97,79%	97,73%	97,75%	99,04%
B. mycoides JAS481	89,95%	99,42%	97,71%	97,72%	99,10%	95,18%	95,13%	97,74%	97,76%	97,72%	99,12%
B. mycoides JAS635	89,99%	97,73%	99,96%	98,47%	97,72%	95,06%	94,96%	98,22%	98,28%	97,83%	97,74%
B. thuringiensis HD1	91,84%	89,99%	89,98%	90,02%	90,03%	89,81%	89,77%	89,98%	89,91%	89,99%	90,02%
B. mycoides BPN07/3	89,74%	95,10%	95,06%	95,08%	95,15%	98,99%	98,10%	95,06%	95,05%	95,08%	95,18%
B. mycoides BPN08/1	89,97%	99,14%	97,62%	97,70%	99,01%	95,10%	95,07%	97,73%	97,72%	97,69%	99,03%
B. mycoides ATCC 6462	89,96%	99,29%	97,72%	97,70%	99,04%	95,19%	95,15%	97,72%	97,70%	97,72%	99,07%
B. mycoides JAS823	89,74%	94,95%	94,88%	94,95%	95,00%	97,28%	97,22%	94,94%	94,93%	94,91%	95,03%
B. mycoides BPN51/1	89,93%	97,87%	98,48%	98,44%	97,83%	95,12%	95,05%	98,19%	98,23%	97,97%	97,84%
B. subtilis str. 168	84,41%	83,00%	83,17%	83,13%	82,99%	83,45%	83,06%	83,02%	83,52%	83,25%	83,01%
B. mycoides JAS06/3	90,02%	97,73%	98,37%	98,48%	97,75%	95,01%	94,99%	98,44%	98,62%	97,87%	97,80%
B. cereus ATCC 10987	100,00%	89,92%	89,97%	89,93%	89,95%	89,73%	89,69%	89,94%	89,88%	89,91%	89,95%
B. mycoides BPN09/1	89,92%	100,00%	97,74%	97,72%	99,03%	95,12%	95,08%	97,75%	97,74%	97,75%	99,00%
B. mycoides JAS1004	89,97%	97,74%	100,00%	98,49%	97,73%	95,07%	94,96%	98,24%	98,29%	97,83%	97,75%
B. mycoides BPN52/2	89,93%	97,72%	98,49%	100,00%	97,71%	95,10%	95,07%	98,52%	98,40%	97,93%	97,75%
B. mycoides JAS85/1	89,95%	99,03%	97,73%	97,71%	100,00%	95,14%	95,08%	97,80%	97,83%	97,71%	99,74%
B. mycoides BPN102	89,73%	95,12%	95,07%	95,10%	95,14%	100,00%	98,11%	95,08%	95,07%	95,08%	95,17%
B. mycoides BPN03/1	89,69%	95,08%	94,96%	95,07%	95,08%	98,11%	100,00%	95,08%	95,01%	95,04%	95,09%
B. mycoides KBAB4	89,94%	97,75%	98,24%	98,52%	97,80%	95,08%	95,08%	100,00%	98,39%	97,83%	97,83%
B. mycoides DSM 11821	89,88%	97,74%	98,29%	98,40%	97,83%	95,07%	95,01%	98,39%	100,00%	97,84%	97,85%
B. mycoides BPN121	89,91%	97,75%	97,83%	97,93%	97,71%	95,08%	95,04%	97,83%	97,84%	100,00%	97,73%
B. mycoides JAS23/1	89,95%	99,00%	97,75%	97,75%	99,74%	95,17%	95,09%	97,83%	97,85%	97,73%	100,00%

Legend:
Bacillus mycoides isolates from Northeastern Poland are marked in black, while the species reference strains are marked in black and given on yellow background. Other *Bacillus cereus s. l.* species references and *Bacillus subtilis* strain 168 are marked in blue.
ANI values higher than ~92.5% proposed by Carroll *et al.* [2020] as a threshold for *B. cereus s.l.* genomospecies, are given on green background, while lower than 92.5% are given on red background. The intensity of the green color increases with the higher ANI value

Table S7. KEGG functional categories in *Bacillus mycoides* isolates under study.

Isolates from Białowieża National Park (n=21)

KEGG level 1	Chromosomal genes		Plasmid genes		Chromosomal and plasmid genes		KEGG level 2	Chromosomal genes		Plasmid genes		Chromosomal and plasmid genes	
	No.	Percent	No.	Percent	No.	Percent		No.	Percent	No.	Percent	No.	Percent
METABOLISM	1 716	48.0	431	45.9	2 147	47.5	09101 Carbohydrate metabolism	418	11.7	123	13.1	541	12
							09102 Energy metabolism	123	3.4	12	1.3	135	3
							09103 Lipid metabolism	56	1.6	24	2.6	80	1.8
							09104 Nucleotide metabolism	115	3.2	23	2.4	138	3.1
							09105 Amino acid metabolism	266	7.4	39	4.1	305	6.8
							09106 Metabolism of other amino acids	44	1.2	8	0.9	52	1.2
							09107 Glycan biosynthesis and metabolism	57	1.6	22	2.3	79	1.7
							09108 Metabolism of cofactors and vitamins	198	5.5	32	3.4	230	5.1
							09109 Metabolism of terpenoids and polyketides	51	1.4	6	0.6	57	1.3
							09110 Biosynthesis of other secondary metabolites	15	0.4	6	0.6	21	0.5
							09111 Xenobiotics biodegradation and metabolism	19	0.5	7	0.7	26	0.6
							09181 Protein families: metabolism	121	3.4	55	5.9	176	3.9
							09191 Unclassified: metabolism	233	6.5	74	7.9	307	6.8
GENETIC INFORMATION PROCESSING	645	18.04	160	17.02	805	17.8	09121 Transcription	6	0.2	1	0.1	7	0.2
							09122 Translation	95	2.7	1	0.1	96	2.1
							09123 Folding, sorting and degradation	48	1.3	7	0.7	55	1.2
							09124 Replication and repair	60	1.7	17	1.8	77	1.7
							09182 Protein families: genetic information processing	379	10.6	108	11.5	487	10.8
							09192 Unclassified: genetic information processing	57	1.6	26	2.8	83	1.8
ENVIRONMENTAL INFORMATION PROCESSING	907	25.36	255	27.1	1 162	25.7	09131 Membrane transport	222	6.2	40	4.3	262	5.8
							09132 Signal transduction	137	3.8	36	3.8	173	3.8
							09183 Protein families: signaling and cellularprocesses	384	10.7	147	15.6	531	11.8
							09193 Unclassified: signaling and cellular processes	164	4.5	32	3.4	196	4.3
CELLULAR PROCESSES	117	3.723	35	3.7	152	3.4	09141 Transport and catabolism	2	0.1			2	0.1
							09143 Cell growth and death	14	0.4	8	0.9	22	0.5
							09145 Cellular community - prokaryotes	70	2	26	2.8	96	2.1
							09142 Cell motility	31	0.9	1	0.1	32	0.7
HUMAN DISEASES	30	0.8	18	1.9	48	1.1	09171 Infectious disease: bacterial	12	0.3	8	0.9	20	0.4
							09175 Drug resistance: antimicrobial	18	0.5	10	1	28	0.6
NOT INCLUDED IN PATHWAY AND BRITE	162	4.5	41	4.4	203	4.5	09194 Poorly characterized	162	4.5	41	4.4	204	4.5

Isolates from a farm in Jasienówka (n=14)

KEGG level 1	Chromosomal genes		Plasmid genes		Chromosomal and plasmid genes		KEGG level 2	Chromosomal genes		Plasmid genes		Chromosomal and plasmid genes	
	No.	Percent	No.	Percent	No.	Percent		No.	Percent	No.	Percent	No.	Percent
METABOLISM	1 680	47.6	319	43.0	1 999	46.8	09101 Carbohydrate metabolism	417	11.8	64	8.6	481	11.3
							09102 Energy metabolism	126	3.6	22	3.0	148	3.5
							09103 Lipid metabolism	56	1.6	11	1.5	67	1.6
							09104 Nucleotide metabolism	111	3.1	16	2.2	127	3.0
							09105 Amino acid metabolism	250	7.1	43	5.8	293	6.9
							09106 Metabolism of other amino acids	43	1.2	6	0.8	49	1.1
							09107 Glycan biosynthesis and metabolism	50	1.4	31	4.2	81	1.9
							09108 Metabolism of cofactors and vitamins	196	5.6	25	3.4	221	5.2
							09109 Metabolism of terpenoids and polyketides	45	1.3	4	0.5	49	1.1
							09110 Biosynthesis of other secondary metabolites	14	0.4	4	0.5	18	0.4
							09111 Xenobiotics biodegradation and metabolism	21	0.6	4	0.5	25	0.6
							09181 Protein families: metabolism	121	3.4	39	5.3	160	3.7
							09191 Unclassified: metabolism	230	6.5	50	6.7	280	6.6
GENETIC INFORMATION PROCESSING	647	18.3	98	13.2	745	17.5	09121 Transcription	7	0.2	0	0	7	0.2
							09122 Translation	94	2.7	2	0.3	96	2.2
							09123 Folding, sorting and degradation	50	1.4	9	1.2	59	1.4
							09124 Replication and repair	63	1.8	16	2.2	79	1.9
							09182 Protein families: genetic information processing	379	10.7	71	9.6	450	10.5
							09192 Unclassified: genetic information processing	54	1.5	0	0	54	1.3
ENVIRONMENTAL INFORMATION PROCESSING	893	25.3	240	32.4	1133	26.5	09131 Membrane transport	217	6.1	33	4.5	250	5.9
							09132 Signal transduction	140	4	38	5.1	178	4.2
							09183 Protein families: signaling and cellularprocesses	372	10.5	169	22.8	541	12.7
							09193 Unclassified: signaling and cellular processes	164	4.6	0	0	164	3.8
CELLULAR PROCESSES	117	3.3	30	3.4	147	3.4	09141 Transport and catabolism	2	0.1	0	0	2	0.1
							09143 Cell growth and death	15	0.4	4	0.5	19	0.4
							09145 Cellular community - prokaryotes	68	1.9	25	3.4	93	2.2
							09142 Cell motility	32	0.9	1	0.1	33	0.8
HUMAN DISEASES	29	0.8	22	3.0	51	1.2	09171 Infectious disease: bacterial	13	0.4	14	1.9	27	0.6
							09175 Drug resistance: antimicrobial	16	0.5	8	1.1	24	0.6
NOT INCLUDED IN PATHWAY AND BRITE	162	4.6	32	4.3	195	4.6	09194 Poorly characterized	162	4.6	32	4.3	195	4.6
